# Supplementary material for: A Bacteroides thetaiotaomicron genetic locus encodes activities consistent with mucin O-glycoprotein processing and N-acetylgalactosamine metabolism
Source: Nat Commun. 2025 Apr 12;16:3485. doi: 10.1038/s41467-025-58660-2 (PMC11992087; doi:10.1038/s41467-025-58660-2)
Supplement: Supplementary file 1 — Supplementary Information [file 41467_2025_58660_MOESM1_ESM.pdf]

Ndeh et al., Supplemental data.

Table S1. Affinity of BT4244 and BT4245 CBM32 domains and BT4246 SusD-like for mucin derived mono- and di-saccharides as derived by ITC.

Protein	Sugar	Ka × 10^4 (M^{-1})
**BT4244-CBM32[a]**	GalNAc	0.40 (±0.03)
	Gal	0.16 (±0.01)
	NeuAc	NB[b]
	Fuc	NB
	GlcNAc	NB
	Lactose	0.23 (±0.00)
	Core 1	0.20 (±0.01)
**BT4245 (SGBP[c])**	GalNAc	1.20 (±0.2)
	Gal	TLTF[d]
	NeuAc	NB
	Fuc	NB
	GlcNAc	NB
	Lactose	0.21 (±0.02)
	Core 1	0.36 (±0.01)
	F antigen	0.88 (±0.06)
**BT4246 (SusD-like)**	GalNAc	TLTF
	Gal	TLTF
	NeuAc	NB
	Fuc	NB
	GlcNAc	NB
	α-Galactobiose	TLTF
	Lactose	TLTF
	LacNAc	TLTF
	LNB	0.13 (±0.07)
	Core 1	0.45 (±0.03)
**BT4244-BACON[e]**	GalNAc	NB
	Gal	NB
	NeuAc	NB
	Fuc	NB
	GlcNAc	NB
	α-Galactobiose	NB
	Lactose	NB
	LacNAc	NB
	LNB	NB
	Core 1	NB

[a] CBM32 alone tested. [b]NB: no binding detected. [c]SGBP: surface glycan binding protein. Full length protein lacking signal peptide was tested. [d]TLTF: weak binding observed but affinity too low to fit (Ka <~0.1×10^4 M^{-1}). [e]N-terminal BACON domain alone tested.
Lactose: Galβ1-4Glc, LacNAc: Galβ1-4GlcNAc, LNB: Galβ1-3GlcNAc Core 1: Galβ1-3GalNAc. α-Galactobiose: Galα1-3Gal, F-antigen (core 5): GalNAcα1-3GalNAc. Where binding was observed the data shown are averages and standard deviations of at least triplicate titrations.

Table S2. Data Collection and Refinement Statistics for BT4246 SusD-like.

	SeMet	Native	Nat/O-glycan
**PDB id**	5CK1	5CK0	5CJZ
**Resolution (Å)**	31.83 - 1.835 (1.901 - 1.835)	46.23 - 1.996 (2.068 - 1.996)	41.88 - 1.803 (1.867 - 1.803)
**Space group**	C 2 2 21	P 6 2 2	P 6 2 2
**Unit cell (a,b,c)**	110.7, 115.7, 116.6	156.2, 156.2, 114.7	155.9, 155.9, 114.3
**Total reflections**	447332 (35783)	264067 (24638)	975782 (80697)
**Unique reflections**	65430 (6379)	55908 (5331)	74235 (7283)
**Multiplicity**	6.8 (5.6)	4.7 (4.6)	13.1 (11.1)
**Completeness (%)**	99.66 (98.08)	99.25 (96.63)	98.25 (98.13)
**Mean I/sigma(I)**	37.41 (13.30)	8.03 (1.66)	10.95 (1.03)
**Wilson B-factor**	16.93	16	21.06
**R-merge**	0.1095 (0.213)	0.2025 (0.881)	0.2301 (1.797)
**R-meas**	0.1185	0.228	0.2392
**CC1/2**	0.986 (0.959)	0.963 (0.315)	0.99 (0.458)
**CCstar**	0.996 (0.99)	0.991 (0.692)	0.997 (0.793)
**R-work**	0.1671 (0.1981)	0.1867 (0.2507)	0.1712 (0.2970)
**R-free**	0.2013 (0.2595)	0.2265 (0.3068)	0.2050 (0.3568)
**non-hydrogen atoms**	5584	5565	5811
**macromolecules**	4752	4861	4861
**ligands**	13	5	30
**water**	819	699	920
**Protein residues**	585	603	603
**RMS(bonds)**	0.006	0.007	0.007
**RMS(angles)**	0.93	0.97	0.92
**Ramachandran favored (%)**	97	97	97
**Ramachandran outliers (%)**	0.17	0.33	0.17
**Clashscore**	1.68	1.91	1.43

**Average B-factor**	19.6	13.2	19.2
**macromolecules**	17.8	12.2	17.3
**ligands**	21.4	25.1	25.5
**solvent**	30.4	19.4	28.9

Table S3. BT4241-GH2, BT4243-GH109 and BT4240-kinase kinetic data.

**Enzyme**	**Substrate**	**k_{cat} (s^{-1})**	**K_M (mM)**	**k_{cat}/K_M (s^{-1} mM^{-1})**
**BT4241-GH2[a]**	Core 1 (Galβ1-3GalNAc)	14.2 (±0.6)	0.36 (±0.04)	39.4
	LNB (Galβ1-3GlcNAc)	6.1 (±0.6)	0.5 (±0.1)	12.2
	LacNAc (Galβ1-4GlcNAc)	0.18 (±0.02)	1.4 (±0.3)	0.13
	Galactobiose (Galβ1-3Gal)	7.0	1.0	7.0
**BT4243-GH109**	PNP-α-GalNAc	2.1 (±0.3)	0.03 (±0.005)	70
	PNP-β-GalNAc[b]	0.5 (±0.0.04)	0.08 (±0.01)	6.3
**BT4240-kinase**	GalNAc	3.0 (±0.04)	0.9 (±0.04)	3.3
	GlcNAc	0.34 (±0.03)	27.5 (±4.8)	0.012

[a]Both GH enzymes were initially screened against a range of PNP-linked monosaccharides (β-Gal, β-Glc, β-Man, α-GalNAc, β-GalNAc, α-GlcNAc, β-GlcNAc, β-L-Fuc, α-L-Fuc, α-Gal and α-Glc) in 20 mM MOPS buffer, pH 7.0. Activity for BT4241-GH2 was only observed with pNP-β-Gal.
Activity for BT4243-GH109 was only observed with PNP-α-GalNAc and PNP-β-GalNAc.
[b]GH109 enzymes have previously been shown to act on both α and β linked substrates due to their unusual NAD-dependent hydrolysis mechanism [1]. Data shown are averages and standard deviations of at least triplicate assays, except for galactobiose which was only run once due to limited substrate availability. Source data are provided as a Source Data file.

Table S4. Additional candidate surface proteases from *B. theta* PULs induced by mucin.

Locus tag of predicted protease	Merops family	Predicted PUL[a]	LipoP prediction[b]
**BT0212**	Subfamily S8 unassigned peptidase (MER028054)	BT0206-14	SpII score=17.8 margin=11.9
**BT3015**	M60L	BT3012-15	SpII score=20.5 margin=2.3
**BT3960**	Subfamily C2A unassigned peptidase (MER003948)	BT3958-61	SpII score=13.7 margin=12.0

[a] *B. theta* PULs upregulated during growth on mucins *in vitro* or *in vivo* [2].

[b] LipoP 1.0 server [3].

Table S5. IBD-TaMMA based comparative metatranscriptomics data.

Stool_Control-vs-Ileum_Control.

Species	log2 FC	log2 FC SE	P-value	FDR	Average abundance
Bacteroides cellulosilyticus	14.8763215	0.24347553	0	0	15714.01043
Bacteroides zoogleoformans	14.5659717	0.24376752	0	0	12194.2136
Bacteroides intestinalis	14.1992032	0.24929319	0	0	9341.715141
Bacteroides sp. A1C1	14.0814107	0.22498263	0	0	19747.02844
Bacteroides uniformis	14.0548091	0.22907847	0	0	71379.11292
Bacteroides xylanisolvens	13.991596	0.22710203	0	0	13388.59627
Bacteroides coprosuis	13.908992	0.21438103	0	0	4614.73619
Bacteroides salanitronis	13.8424973	0.25163476	0	0	5047.158573
Bacteroides vulgatus	13.4880708	0.263783	0	0	234095.4615
Bacteroides heparinolyticus	13.1040165	0.22329803	0	0	6360.181734
Bacteroides helcogenes	12.8696676	0.22725144	0	0	4510.868548
Bacteroides dorei	12.3734365	0.26344534	0	0	57548.0707
Bacteroides ovatus	12.1824643	0.22804189	0	0	32555.42631
Bacteroides caecimuris	12.1113107	0.21501407	0	0	2749.736735
Bacteroides thetaiotaomicron	11.7090632	0.23941326	0	0	20350.42659
Bacteroides sp. CBA7301	11.2061592	0.21009713	0	0	1934.044617
Bacteroides caccae	11.0011456	0.22456888	0	0	27116.03381
Bacteroides fragilis	10.9077995	0.2597589	0	0	57896.77156

Stool_CD-vs-Stool_control

Species	log2 FC	log2 FC SE	P-value	FDR	Average abundance
Bacteroides fragilis	2.33670678	0.20993824	8.92E-29	2.13E-27	57896.7716
Bacteroides coprosuis	-1.5805525	0.15236942	3.28E-25	6.48E-24	4614.73619
Bacteroides xylanisolvens	1.09228155	0.16345109	2.35E-11	1.77E-10	13388.5963
Bacteroides dorei	-1.3130867	0.21362386	7.91E-10	5.12E-09	57548.0707
Bacteroides ovatus	1.03238852	0.18280092	1.63E-08	9.05E-08	32555.4263
Bacteroides salanitronis	-0.9190471	0.18567494	7.43E-07	3.40E-06	5047.15857
Bacteroides sp. A1C1	0.7084345	0.16907275	2.79E-05	0.00010303	19747.0284
Bacteroides intestinalis	-0.7301931	0.18535722	8.17E-05	0.00028068	9341.71514
Bacteroides cellulosilyticus	-0.6638668	0.1823749	0.00027251	0.00085756	15714.0104
Bacteroides heparinolyticus	-0.4485985	0.16736903	0.00735571	0.01726319	6360.18173
Bacteroides uniformis	0.45893191	0.1825107	0.01191851	0.02644865	71379.1129
Bacteroides thetaiotaomicron	0.40473959	0.19242173	0.03543094	0.06763721	20350.4266
Bacteroides caecimuris	0.29240365	0.15263574	0.05540383	0.0972189	2749.73674
Bacteroides helcogenes	-0.3023877	0.1652247	0.06722596	0.11400667	4510.86855
Bacteroides sp. CBA7301	0.25777077	0.15137516	0.08859418	0.14298861	1934.04462
Bacteroides caccae	0.28077877	0.18148986	0.12184471	0.18576872	27116.0338
Bacteroides vulgatus	-0.0511873	0.21394109	0.81090497	0.85616399	234095.461
Bacteroides zoogleoformans	-0.0240906	0.17841245	0.89258995	0.92087949	12194.2136

Stool_UC-vs-Stool_Control

Species	log2 FC	log2 FC SE	P-value	FDR	Average abundance
Bacteroides coprosuis	-1.7909021	0.16748618	1.10E-26	3.15E-25	4614.73619
Bacteroides salanitronis	-1.8192606	0.20409655	4.93E-19	8.25E-18	5047.15857
Bacteroides fragilis	1.77603516	0.23076518	1.40E-14	1.62E-13	57896.7716
Bacteroides xylanisolvens	-0.890205	0.17966751	7.24E-07	3.88E-06	13388.5963
Bacteroides dorei	-1.1514826	0.23481648	9.40E-07	4.95E-06	57548.0707
Bacteroides zoogleoformans	-0.9361744	0.19611244	1.81E-06	9.11E-06	12194.2136
Bacteroides cellulosilyticus	-0.8266727	0.20046757	3.73E-05	0.00015293	15714.0104
Bacteroides uniformis	0.56283307	0.20061671	0.00502363	0.01320399	71379.1129
Bacteroides caecimuris	-0.4215706	0.16777977	0.01198307	0.02868727	2749.73674
Bacteroides sp. CBA7301	0.34596829	0.16639181	0.03759541	0.07705061	1934.04462
Bacteroides heparinolyticus	-0.3568871	0.1839729	0.05239314	0.10267663	6360.18173
Bacteroides caccae	0.34334451	0.19949459	0.08523779	0.1557205	27116.0338
Bacteroides intestinalis	-0.3202277	0.20374544	0.11601898	0.19979497	9341.71514
Bacteroides ovatus	0.18139242	0.20093584	0.36666495	0.48460094	32555.4263
Bacteroides helcogenes	0.12662401	0.18161533	0.48567145	0.59585682	4510.86855
Bacteroides sp. A1C1	-0.129103	0.18584588	0.48725718	0.59677993	19747.0284
Bacteroides thetaiotaomicro	0.06055923	0.21151105	0.77463525	0.83672624	20350.4266
Bacteroides vulgatus	0.03680001	0.23516518	0.87565012	0.91242684	234095.461

Colon_CD-vs-Colon_Control

Species	log2 FC	log2 FC SE	P-value	FDR	Average abundance
Bacteroides cellulosilyticus	-1.1576731	0.38989476	0.00298582	0.04972731	15714.0104
Bacteroides uniformis	-1.0410075	0.35781566	0.00362192	0.05738644	71379.1129
Bacteroides vulgatus	-1.1203728	0.40045115	0.00514557	0.07390924	234095.461
Bacteroides dorei	-0.9088947	0.40356843	0.0243131	0.22264452	57548.0707
Bacteroides intestinalis	-0.8773281	0.40288597	0.02943516	0.25180625	9341.71514
Bacteroides coprosuis	-0.5753001	0.33924516	0.0899192	0.49973942	4614.73619
Bacteroides zoogleoformans	0.59093229	0.37771044	0.11769751	0.58475483	12194.2136
Bacteroides thetaiotaomicror	-0.5056922	0.38748881	0.19187584	0.74621692	20350.4266
Bacteroides xylanisolvens	0.45571599	0.35706842	0.20185981	0.76027162	13388.5963
Bacteroides sp. A1C1	-0.3988204	0.36012966	0.26810565	0.8487108	19747.0284
Bacteroides heparinolyticus	-0.3730743	0.37140499	0.31514025	0.88895238	6360.18173
Bacteroides salanitronis	0.28235526	0.37469634	0.45111493	0.98386891	5047.15857
Bacteroides helcogenes	-0.2128452	0.35109928	0.54436524	0.99788135	4510.86855
Bacteroides sp. CBA7301	0.19042384	0.3391709	0.57449817	0.99788135	1934.04462
Bacteroides caecimuris	0.18447228	0.34399084	0.59177133	0.99788135	2749.73674
Bacteroides caccae	0.15260376	0.36962756	0.67971052	0.99788135	27116.0338
Bacteroides fragilis	-0.0788028	0.4055524	0.84593342	0.99788135	57896.7716
Bacteroides ovatus	0.03932813	0.35518841	0.91183465	0.99788135	32555.4263

Colon_UC-vs-Colon_Control

Species	log2 FC	log2 FC SE	P-value	FDR	Average abundance
Bacteroides salanitronis	1.75569144	0.35483292	7.50E-07	2.39E-05	5047.15857
Bacteroides zoogleoformans	1.64123234	0.35749919	4.41E-06	0.00011833	12194.2136
Bacteroides coprosuis	0.98935797	0.30795871	0.00131525	0.01708118	4614.73619
Bacteroides ovatus	-1.1201346	0.35591391	0.00164838	0.02086705	32555.4263
Bacteroides vulgatus	0.94060667	0.38633959	0.01490567	0.11287329	234095.461
Bacteroides helcogenes	0.58088736	0.33146978	0.07969483	0.31935265	4510.86855
Bacteroides sp. CBA7301	0.55346605	0.32482849	0.08840507	0.33771935	1934.04462
Bacteroides heparinolyticus	0.54862071	0.3457749	0.11259393	0.38911765	6360.18173
Bacteroides sp. A1C1	0.43920907	0.33878817	0.19483356	0.52567312	19747.0284
Bacteroides caccae	0.45346926	0.35657311	0.20346454	0.5393801	27116.0338
Bacteroides uniformis	-0.4012628	0.34276745	0.24173699	0.58457672	71379.1129
Bacteroides fragilis	0.2839096	0.39201218	0.46892044	0.78879172	57896.7716
Bacteroides caecimuris	0.21019276	0.33528247	0.53071662	0.82958603	2749.73674
Bacteroides dorei	0.23570502	0.38880954	0.54436714	0.83663698	57548.0707
Bacteroides thetaiotaomicron	0.18930915	0.36958993	0.60850123	0.86469861	20350.4266
Bacteroides xylanisolvens	0.09926204	0.35483367	0.7796751	0.9332579	13388.5963
Bacteroides cellulosilyticus	-0.0578602	0.36063106	0.87253321	0.95865884	15714.0104
Bacteroides intestinalis	-0.0012612	0.37520843	0.99731798	0.99924642	9341.71514

Ileum_UC-vs-Ileum_Control

Species	log2 FC	log2 FC SE	P-value	FDR	Average abundance
Bacteroides thetaiotaomicron	8.76966677	0.26119922	3.94E-247	8.62E-245	20350.4266
Bacteroides uniformis	6.81763364	0.24961027	2.96E-164	2.60E-162	71379.1129
Bacteroides vulgatus	7.78648422	0.28820416	9.27E-161	7.70E-159	234095.461
Bacteroides dorei	7.53416535	0.28782648	4.98E-151	3.53E-149	57548.0707
Bacteroides sp. A1C1	5.98972484	0.24322011	6.53E-134	3.84E-132	19747.0284
Bacteroides sp. CBA7301	5.38338267	0.22610472	2.68E-125	1.43E-123	1934.04462
Bacteroides ovatus	5.79881173	0.24873749	3.27E-120	1.61E-118	32555.4263
Bacteroides caecimuris	5.34877566	0.23102442	1.37E-118	6.43E-117	2749.73674
Bacteroides xylanisolvens	5.35356692	0.24439302	2.30E-106	9.08E-105	13388.5963
Bacteroides intestinalis	5.78375735	0.2691788	2.07E-102	7.63E-101	9341.71514
Bacteroides salanitronis	5.56356462	0.27151169	2.59E-93	8.31E-92	5047.15857
Bacteroides coprosuis	4.63284754	0.23076564	1.20E-89	3.72E-88	4614.73619
Bacteroides helcogenes	4.82286459	0.24507891	3.28E-86	9.86E-85	4510.86855
Bacteroides fragilis	5.29065795	0.28368	1.26E-77	3.39E-76	57896.7716
Bacteroides cellulosilyticus	4.7728082	0.26347167	2.42E-73	6.31E-72	15714.0104
Bacteroides zoogleoformans	3.43799556	0.26435424	1.14E-38	1.69E-37	12194.2136
Bacteroides heparinolyticus	2.64136528	0.24364635	2.20E-27	2.45E-26	6360.18173
Bacteroides caccae	2.38356758	0.24545672	2.71E-22	2.56E-21	27116.0338

Ileum_CD-vs-Ileum_Control

Species	log2 FC	log2 FC SE	P-value	FDR	Average abu
Bacteroides caccae	-3.6412135	0.19008156	8.62E-82	4.98E-79	27116.0338
Bacteroides dorei	-2.7566329	0.2193635	3.23E-36	4.52E-34	57548.0707
Bacteroides ovatus	-2.3602375	0.19359605	3.45E-34	4.69E-32	32555.4263
Bacteroides thetaiotaomicron	-1.9451521	0.20155522	4.88E-22	4.34E-20	20350.4266
Bacteroides vulgatus	-2.0427223	0.21917482	1.16E-20	9.78E-19	234095.462
Bacteroides uniformis	-1.768015	0.19478796	1.12E-19	8.93E-18	71379.1129
Bacteroides heparinolyticus	-0.844399	0.19757203	1.92E-05	0.00062998	6360.18173
Bacteroides sp. A1C1	-0.6801244	0.19802977	0.00059375	0.0137274	19747.0284
Bacteroides fragilis	-0.6800739	0.21615674	0.00165403	0.03429696	57896.7716
Bacteroides sp. CBA7301	-0.4608381	0.18885521	0.01468051	0.2101631	1934.04462
Bacteroides intestinalis	-0.4257251	0.21987652	0.0528431	0.51899459	9341.71514
Bacteroides coprosuis	-0.3471708	0.19359805	0.07293203	0.62946582	4614.73619
Bacteroides helcogenes	-0.310876	0.20259772	0.12491907	0.78269073	4510.86855
Bacteroides zoogleoformans	-0.2749082	0.21637644	0.20390336	0.94379753	12194.2136
Bacteroides cellulosilyticus	-0.2592645	0.21319983	0.22396073	0.96155469	15714.0104
Bacteroides caecimuris	-0.2330882	0.1937831	0.22904204	0.96897564	2749.73674
Bacteroides xylanisolvens	-0.1280325	0.20282377	0.52787721	0.99842806	13388.5963
Bacteroides salanitronis	-0.1142208	0.22174404	0.60648187	0.99842806	5047.15857

Statistical differences between groups were calculated by analysis of variance with Tukey's honestly significant difference post hoc test for multiple comparisons. This corresponds to a two-sided test. Differences with adjusted $P \leq 0.05$ are considered significant[4].

Table S6. Primers used in this study.

Name	Protein id/genetic modification	RE	Sequence (5'-3')
**Primers for cloning into *E. coli* expression vectors**			
**BT4240for**	Kinase	BamHI	CGCGGGATCCATGAAAGATTTATCAA GTATTGTAGC
**BT4240rev**	Kinase	XhoI	CCGGCTCGAG TTATCCATTAACCAAGCACTCATTG
**BT4241for**	GH2	BamHI	CGCGGGATCC ATGGCCGAAAAGACATCCGACAA
**BT4241rev**	GH2	XhoI	CCGGCTCGAG TTATCGATAATCATATTTGGCGGC
**BT4243for**	GH109	BamHI	CGCGGGATCC CAAAAGACAAAAGCAAAGTTCTCT
**BT4243rev**	GH109	XhoI	CCGGCTCGAG TTATTCGGCAAAAGCATGTCTGTA
**BT4244-FLfor**	M60L-full length	BamHI	CGCG GGATCC AAG GAT ACC GAA AAA TCG ATT ATA
**BT4244-FLrev**	M60L-full length	EcoRI	CCGG GAATTC TTA TAA CAG AAT ACG TTT TCC GTC
**BT4245for**	SGBP	NcoI	CGCGCCATGGACAATTATGACGATAC CTATCC
**BT4245rev**	SGBP	XhoI	CCGGCTCGAGTTCGGACAGTATGAA CAGACT

**BT4246for**	SusD-like	Nhe1	GCGATCGCTAGCGATTATCTAGACGT CGTTCCACC
**BT4246rev**	SusD-like	Xho1	GCGATCCTCGAGTTAATATCCGGGTG CTTGTACC
**BT4244-CBM32for**	CBM32 from M60L	NcoI	CGCG CCATGG ACATCAAGGTTACACCAACC
**BT4244-CBM32rev**	CBM32 from M60L	XhoI	CCGG CTCGAG CGTATTTGTTTTGTAAAATTCCATTTC
**BT4244-BCNfor**	BACON domain from M60L	BamHI	CGCG GGATCC AAG GAT ACC GAA AAA TCG ATT ATA
**BT4244-BCNrev**	BACON domain from M60L	EcoRI	CCGG GAATTC TTAGCCTCCGGTTGGTGTAAC
**BT4244-BCN CBM32 for**	BACON-CBM32	BamHI	CGCG GGATCC AAG GAT ACC GAA AAA TCG ATT ATA
**BT4244-BCN-CBM32rev**	BACON-CBM32	EcoRI	CCGG GAATTC TTA CGT ATT TGT TTT GTA AAA TTC CAT TTC
**Primers for genetic manipulation of Bt**			
**Δ4240-50F1for**	BT4240-50 deletion	BamHI	CGCGGGATCCGGATCGATTTCAAGC ATAACAAAT
**Δ4240-50F1rev**	BT4240-50 deletion	None	ATGGGTATGAATACGTTTGAGCCTTG GAGAATGGAAAATGGATAATTAGT
**Δ4240-50F2for**	BT4240-50 deletion	None	ACTAATTATCCATTTTCCATTCTCCAA GGCTCAAACGTATTCATACCCAT
**Δ4240-50F2rev**	BT4240-50 deletion	XbaI	CGCGTCTAGAGTATCTTCATTTACCA CAGTATGGAT
**ΔBT4244F1for**	BT4244 deletion	BamHI	CGCGGGATCCCATGAGTTGCATATAC ATCACCAC
**ΔBT4244F1rev**	BT4244 deletion	None	TCTTAAAAACTAATACAGCAAAAAA GAATAATTTAACTTAACACACATTAC
**ΔBT4244F2for**	BT4244 deletion	None	GTAATGTGTGTTAAGTTAAATTATTCT TTTTTGCTGTATTAGTTTTTAAGA
**ΔBT4244F2rev**	BT4244 deletion	XbaI	CGCGTCTAGACTCTTGAAGAGGGAC AAACGTG
**BT4240-flagF1**	Flag-tagging BT4240	BamHI	CGCGGGATCCATGAAAGATTTATCAA GTATTGTAGC
**BT4240-flagF2**		None	GACTACAAAGACGATGACGACAAAT AATGGAGAATGGAAAATGGATAATTA G
**BT4240-flagR1**		None	TTATTTGTCGTCATCGTCTTTGTAGTC TCCATTAACCAAGCACTCATTG
**BT4240-flagR2**		XbaI	CGCGTCTAGATCAGCGGATTGTATGA CAAGTTTC
**BT4241-flagF1**	Flag-tagging BT4241	BamHI	CGCGGGATCCATTACAAACTCAAAC CCAAGGAG
**BT4241-flagF2**		None	GACTACAAAGACGATGACGACAAAT AACCTTAGTATAAATTTTAAAGAAAA GAC
**BT4241-flagR1**		None	TTATTTGTCGTCATCGTCTTTGTAGTC TCGATAATCATATTTGGCGGCTT
**BT4241-flagR2**		XbaI	CGCGTCTAGACTACGCTTTGAAGTAG TTTGAAC
**BT4243-flagF1**	Flag-tagging BT4243	BamHI	CGCGGGATCCACTCAGATTGTAGCTT TATGCG

**BT4243-flagF2**		None	GACTACAAAGACGATGACGACAAATAAGCCATTATACCTTATTAATATATAAAC
**BT4243-flagR1**		None	TTATTTGTCGTCATCGTCTTTGTAGTCTTCGGCAAAAGCATGTCTGTA
**BT4243-flagR2**		XbaI	CGCGGCGGCCGCCAGTAATAGGTGATATGATCTGTAT
**BT4245-flagF1**	Flag-tagging BT4245	BamHI	CGCGGGATCCGACAATTATGACGATACCTATCC
**BT4245-flagF2**		None	GACTACAAAGACGATGACGACAAATAATAAATGAGAGACAAAGGGTGAGT
**BT4245-flagR1**		None	TTATTTGTCGTCATCGTCTTTGTAGTCTTCGGACAGTATGAACAGACTAAA
**BT4245-flagR2**		XbaI	CGCGTCTAGATGTTGACTCCGGGATTTAGCATA
**Tag1 for**	Signature tag-1	None	ATGTCGCCAATTGTCACTTTCTCA
**Tag11 for**	Signature tag-11	None	ATGCCGCGGATTTATTGGAAGAAG
**Tag rev**		None	CACAATATGAGCAACAAGGAATCC
**NBU2att1for**		None	CCTTTGCACCGCTTTCAACG
**NBU2att1rev**		**None**	TCAACTAAACATGAGATACTAGC

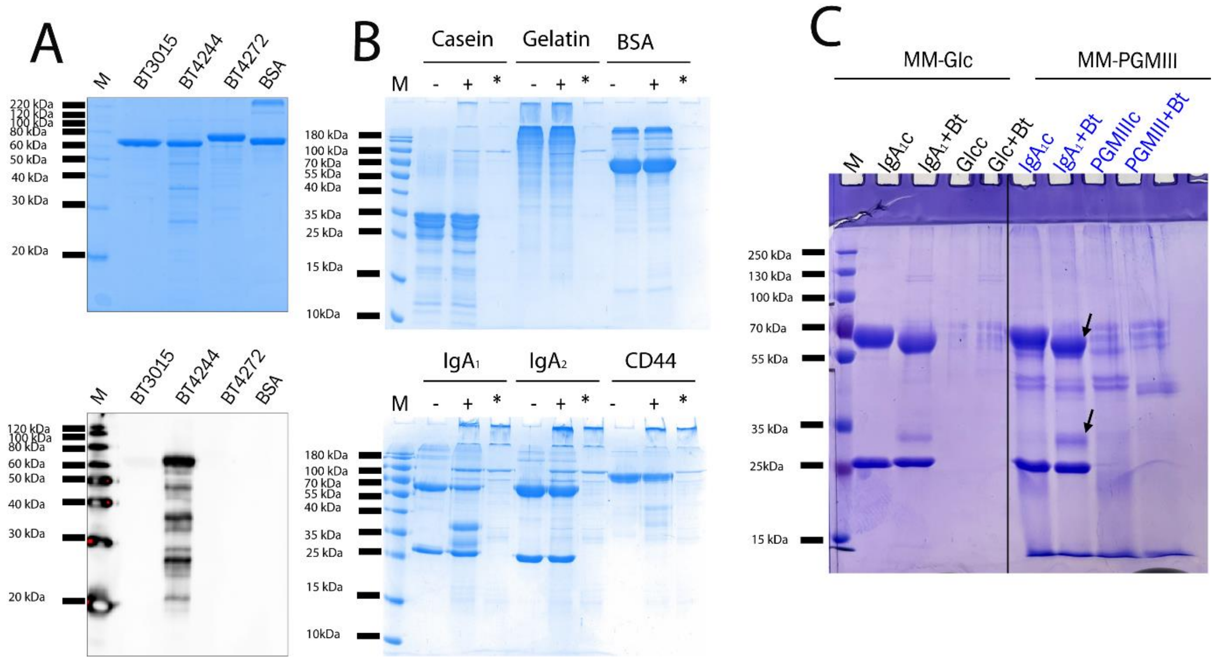

Supplemental Figure 1: Specificity of anti BT4244-M60L polyclonal antibodies and activity of BT4244-M60L and *B. theta* cells against various glycoproteins. **A**: Specificity of anti BT4244-M60L polyclonal antibodies. Upper panel shows SDS-PAGE of recombinant M60-like domains of BT4244 and homologues from *B. theta* (BT3015 and BT4272). The lower panel shows a western blot of the upper panel samples after probing with polyclonal antibodies against the M60-like domain of BT4244-M60L. **B:** SDS-PAGE showing activity of recombinant BT4244-M60L against various potential protein substrates. The -/+ signs represent putative substrate without and with addition of BT4244-M60L enzyme. The lanes marked * are recombinant BT4244-M60L alone without substrate. **C:** SDS-PAGE showing degradation of IgA_1 by *B. theta* cells after culturing in minimal media (MM) containing PGMIII or glucose (Glc). Samples were taken at ~late exponential phase. <u>IgA_1c</u> - Control IgA_1 in MM-Glc or MM-PGMIII; <u>IgA_1+Bt</u> - IgA_1 in MM plus *B. theta* cells and glucose or PGMIII; <u>Glcc</u> – MM-Glc control; <u>Glc+Bt</u> - MM-Glc plus *B. theta* cells; <u>PGMIIIc</u> – MM-PGMIII; <u>PGMIII+Bt</u> – MM-PGMIII plus *B. theta* cells. The lower arrow shows the position of the cleaved IgA heavy chain (HC). The upper arrow shows another processed form of IgA HC that runs at a slightly lower MW than in the control. This appears likely to be due to deglycosylation of the immunoglobulin (either N- or O-deglycosylation or both) by the *B. theta* cells. Notably this doesn't occur with BT4244-M60L alone (see panel B).

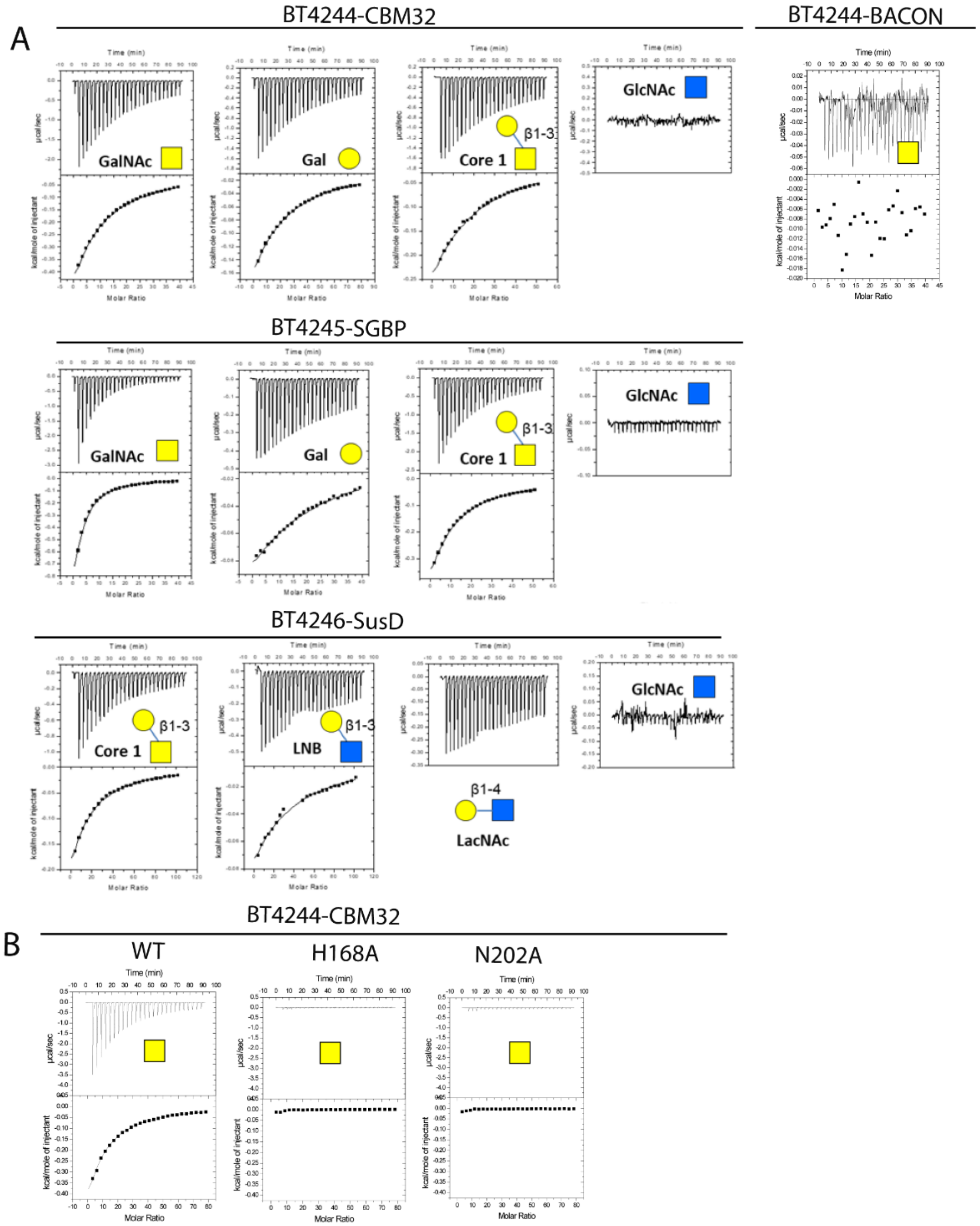

Supplemental Figure 2: Example ITC traces showing specificity of PUL BT4240-50 carbohydrate binding proteins. A: BT4244-CBM32 alone, BT4244-BACON alone, full length SGBP (BT4245; DUF1735-CBM32) and SusD-like (BT4246) vs mucin derived mono- and di-saccharides. **B:** Effect of BT4244-CBM32 point mutations on GalNAc binding.

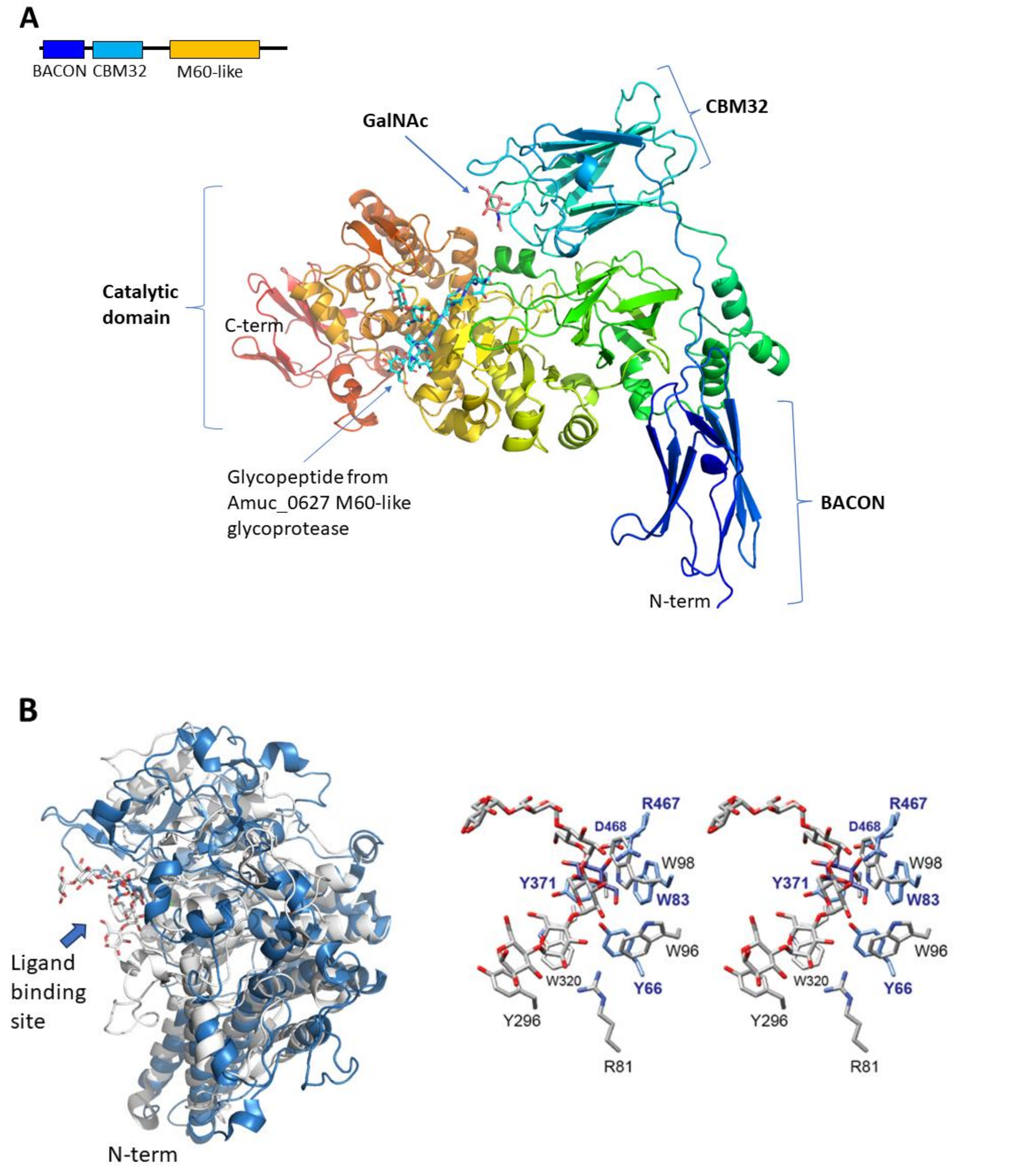

Supplemental Figure 3: Structure of full length BT4244-M60L showing relative position of the CBM32 and comparison of BT4246 SusD-like with the canonical starch binding SusD, BT3701. **A:** Alphafold2 structure (ColabFold v1.5.5) of full length BT4244 (lacking signal peptide) showing the relative positioning of the various domains (colour coded blue to red N- to C-terminus). Yellow coloured region is the M60-like catalytic domain. Cyan sticks show mucin-like glycopeptide modelled from the structure of *Akkermansia muciniphila* M60-like glycoprotease, Amuc_0627 (PDB 7YX8). The carbohydrate binding site of the BT4244-CBM32 is oriented such that the CBM could bind to the glycopeptide substrate in the active site of the catalytic domain, potentially contributing to substrate specificity or aiding in substrate positioning in the active site. The CBM32 is shown with GalNAc (sticks) in its binding site, modelled from an overlay with CpCBM32 (PDB 4AAX). Top left shows relative position of domains in primary sequence. **B:** Left hand of panel is superposition of BT4246

SusD-like (blue) with galactose bound (PDB 5CJZ) and the canonical starch-binding SusD, BT3701 (white) bound to maltohexaose (PDB 3CK9). Overlay of the Cα backbones of both proteins demonstrate that the ligand-binding site is located in the same position on both proteins, though the chemistry of the site is specific for each glycan. Right hand panel shows a stereoview close up of the binding site on BT3701 [5] of the ligand maltohexaose (shown as white sticks with black residue labels) and galactose site of BT4246 (blue sticks, with galactose in dark blue, and blue residue labels).

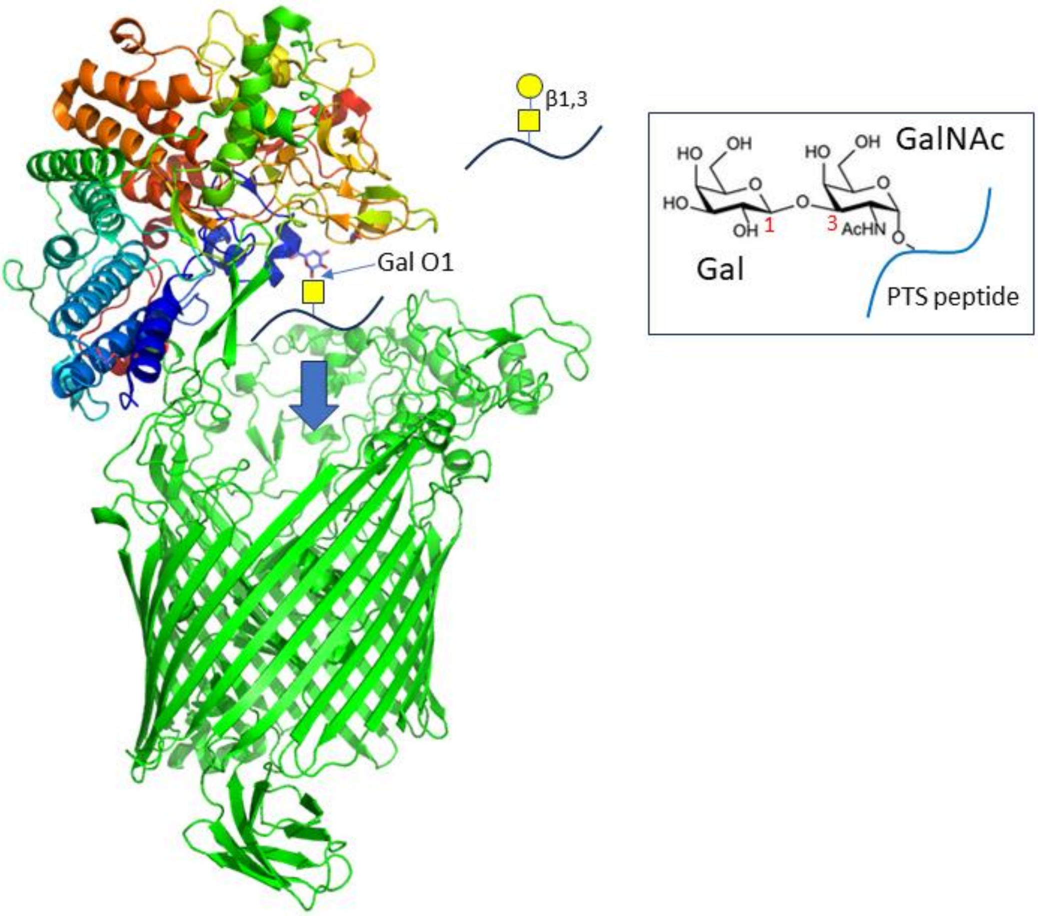

Supplemental Figure 4: Model of BT4246-BT4247 SusCD complex with bound core 1 peptide. The BT4246 SusD-like 'lid' bound to Gal (Gal shown in blue sticks; PDB 5CJZ; colour coded blue to red N- to C- terminus) is shown on top of the Alphafold model of BT4247 SusC-like TonB dependent transporter (green). The BT4246-BT4247 model, using our crystal structure of BT4246 and an Alphafold 3 model of BT4247, was created based upon an overlay of each structure with PDB 6ZLT, which features the open conformation of the SusCD-like proteins BT1762 and BT1763. The O1 of Gal points out of the SusD binding site as seen in the complex with mucin derived *O*-glycans and in this model is attached to the O3 of GalNAc and then a short peptide i.e. a core 1 glycopeptide product of mucin degradation by BT4244-M60L. The core 1 glycopeptide structure that is one of the proposed substrates for the SusCD is also shown fully in symbols at top right for clarity (yellow circle, Gal; yellow square GalNAc; black line short peptide) as well as the actual sugar structures with attached peptide (boxed; peptide shown as blue line). The blue arrow shows the direction of transport for the glycopeptide from the cell surface to the periplasm for deglycosylation. While recognition of a terminal Gal configured sugar could provide a mechanism for selection of a range of glycopeptides substrates, there may also be additional interactions between the SusC and/or SusD and the peptide that could also contribute to selection of the preferred glycopeptide substrates.

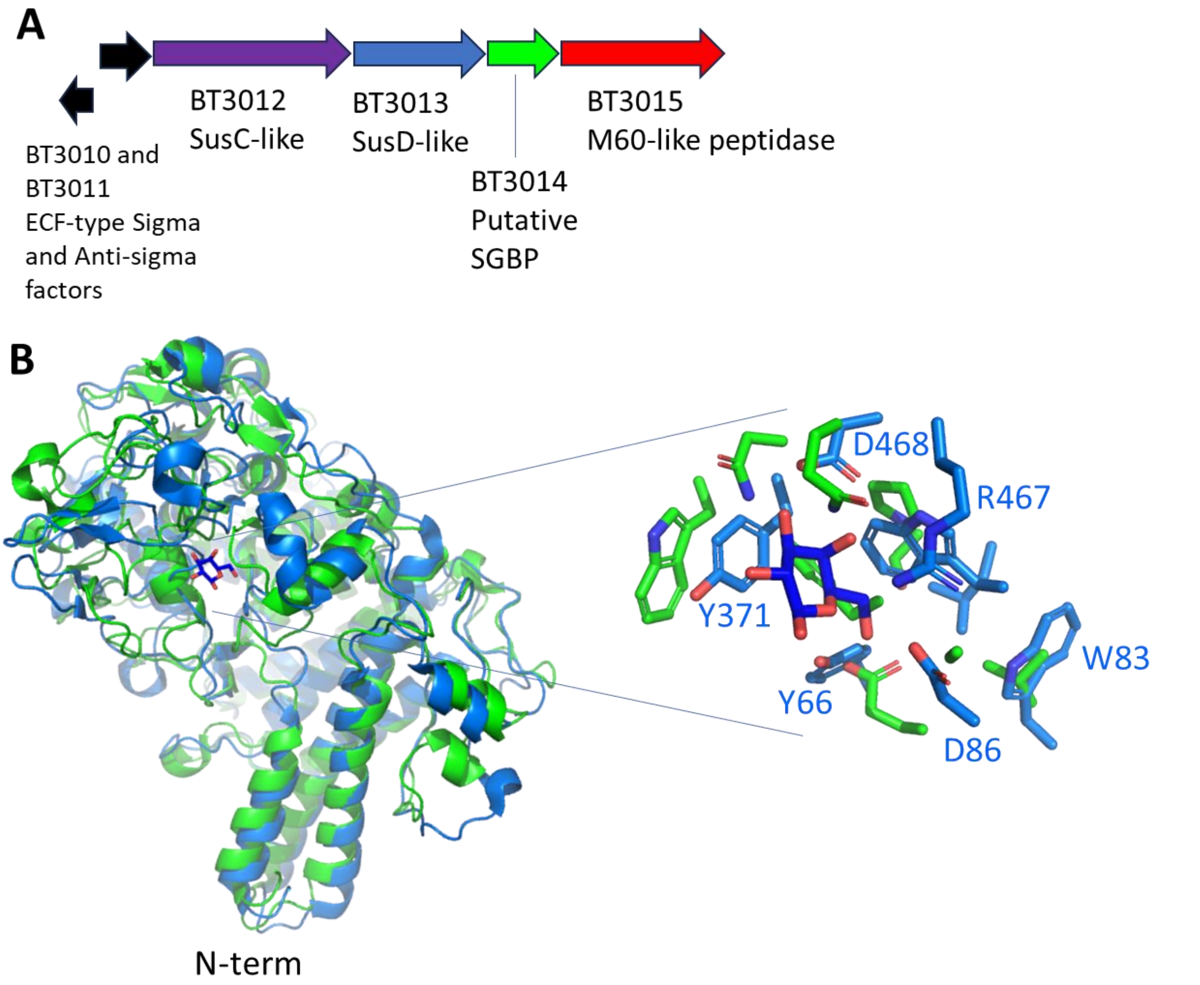

Supplemental Figure 5: Comparison of BT4246 SusD-like with its closest structural homologue BT3013. A: Schematic of syntenic PUL BT3010-3015 (syntenic with BT4244-4250; Fig 2A) of unknown function that contains BT3013, the closest structural homologue of BT4246 SusD-like. The BT3013 PUL is also upregulated during growth on mucins but has no associated CAZymes [2]. **B:** Left of panel shows overlay of BT4246 SusD-like (blue) with galactose bound (blue sticks, PDB 5CJZ) with BT3103 (PDB 7BLL; Z-score = 39.0, 2.4 rmsd Ca, 34% identity). The right-hand panel shows the lack of conservation of the residues in the Gal binding site of BT4246 compared to BT3013, suggesting the latter SusD binds a different ligand to BT4246. Galactose is shown in blue sticks and the residues in BT4246 that interact with the sugar are labelled in blue. The residues in BT3013 in the same region are shown as green sticks.

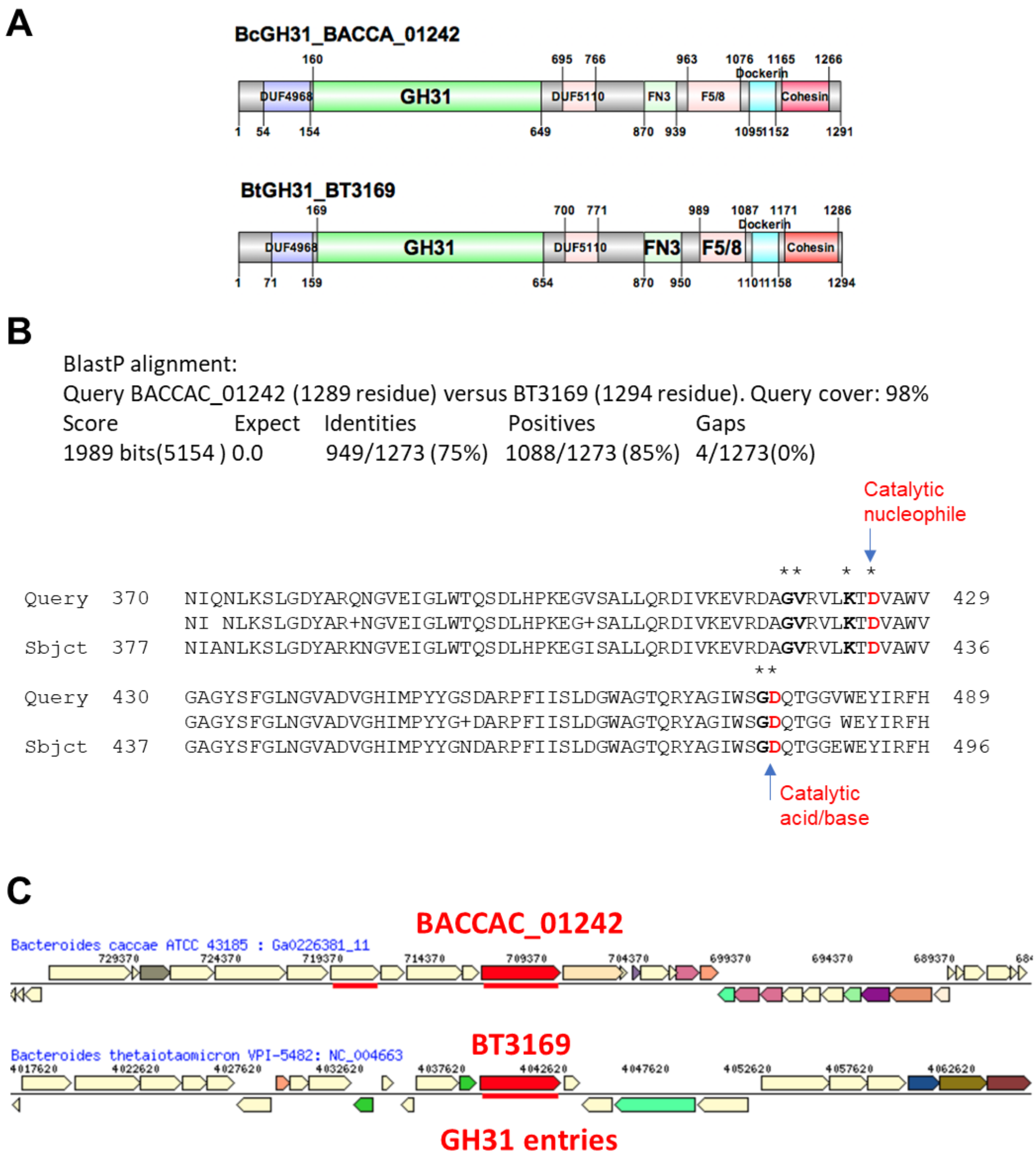

Supplemental Figure 6: Comparison of the GH31 α-N-acetylgalactosaminidases from *B. caccae* with the most similar homologue from *B. theta*. A: Comparison of the protein domain organisation of BACCAC_01242 and BT3169 GH31s using DOG2.0 [6]. **B:** BlastP alignment between BACCAC_01242 and BT3169 GH31s (75% overall identity between the two proteins) focusing on the segments encompassing the key functional residues as defined for BACCAC_01242 [7]. The two residues in red correspond the two key catalytic residues and the stars indicate additional residues considered important for function [7]. **C:** Comparison of the gene neighbourhood of the two most similar GH31s from *B. caccae* (BACCAC_01242) and *B. theta* (BT3169) highlighting their distinct, unrelated, genomic configurations. The figure was generated with the "Gene Cart Neighborhoods" tool at the IMG database (https://img.jgi.doe.gov/cgi-bin/w/main.cgi).

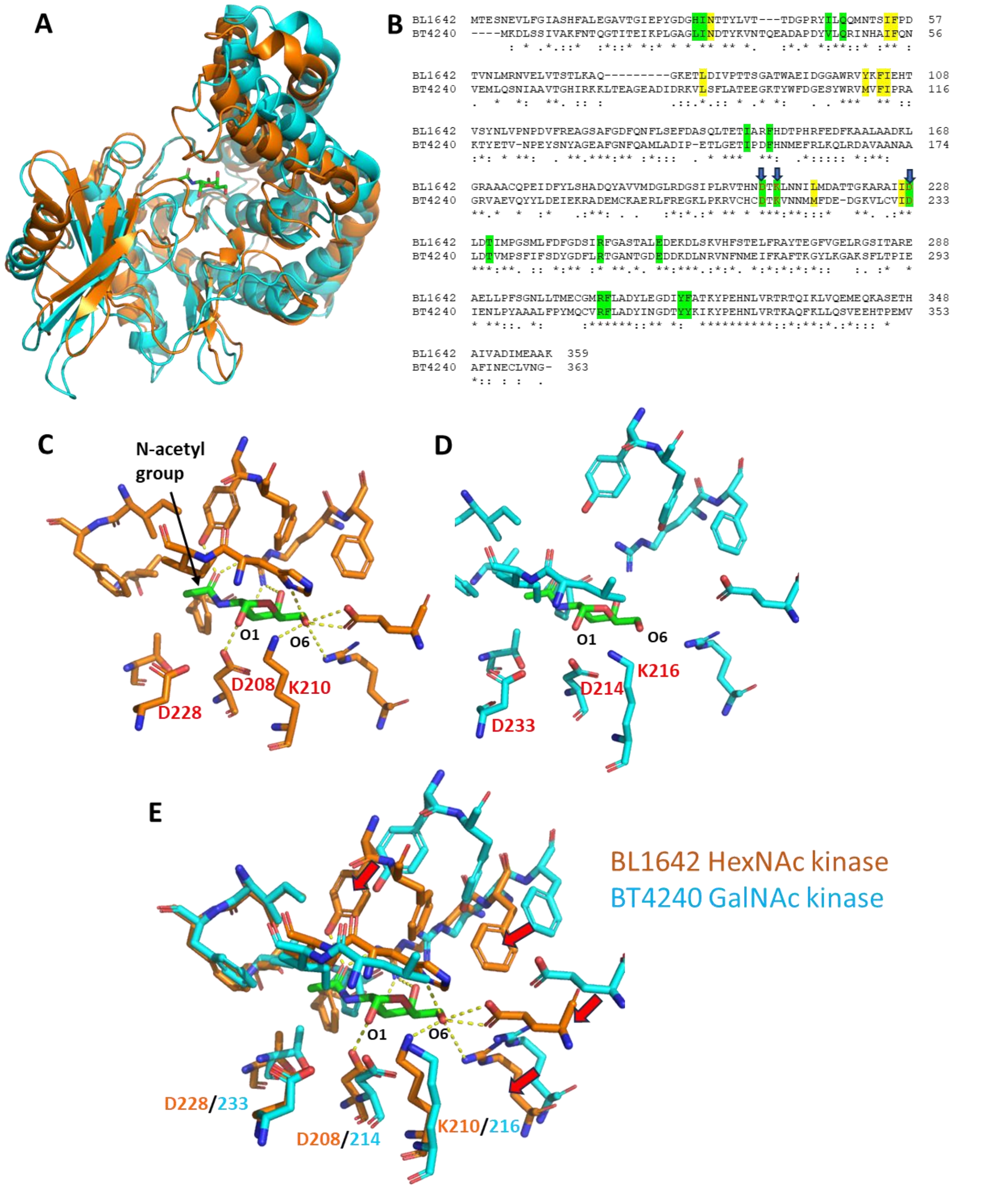

Supplemental Figure 7: Comparison of the structure of the *Bifidobacterium longum* HexNAc kinase BL1642 with the Alphafold model of BT4240 GalNAc kinase. A: Overlay of the structures of the BL1642 HexNAc kinase (orange) with GalNAc bound (green sticks, PDB 4OCQ [8]) with the Alphafold model of BT4240 GalNAc kinase (cyan). While the overall folds are very similar it is clear there are some differences in the positions of the loops and other structural elements around the sugar binding site. This is likely caused by a conformational change in BL1642 where the protein closes around the sugar on binding [8]. The same thing likely happens in BT4240, but the AF model is in the apo ‘open’ form (see also panel E below). **B:** Alignment of BL1642 with BT4240 showing the conservation of the substrate binding residues. Amino acids that comprise the sugar binding site in BL1642 (polar and non-polar interactions) are highlighted in green, while those comprising the ATP binding site are highlighted in yellow. The key catalytic residues are indicated with arrows and are

coloured red [8]. **C:** HexNAc binding site of BL1642 showing the main direct polar and non-polar interactions with the sugar (GalNAc). H-bonds are shown as yellow dotted lines. The key catalytic residues involved at phosphorylation at O1 are labelled. **D:** Predicted BT4240 GalNAc binding site based on the overlay with BL1642, showing the conservation of residues interacting with the sugar including the key catalytic residues (labelled). GalNAc is from the BL1642 structure. **E:** Overlay of the BL1642 and BT4240 sugar binding sites. Red arrows show the likely direction of movement of the residues in BT4240 on GalNAc binding from the apo 'open' to the sugar bound 'closed' state. The ATP binding site is not shown for clarity but is highly conserved in both proteins.

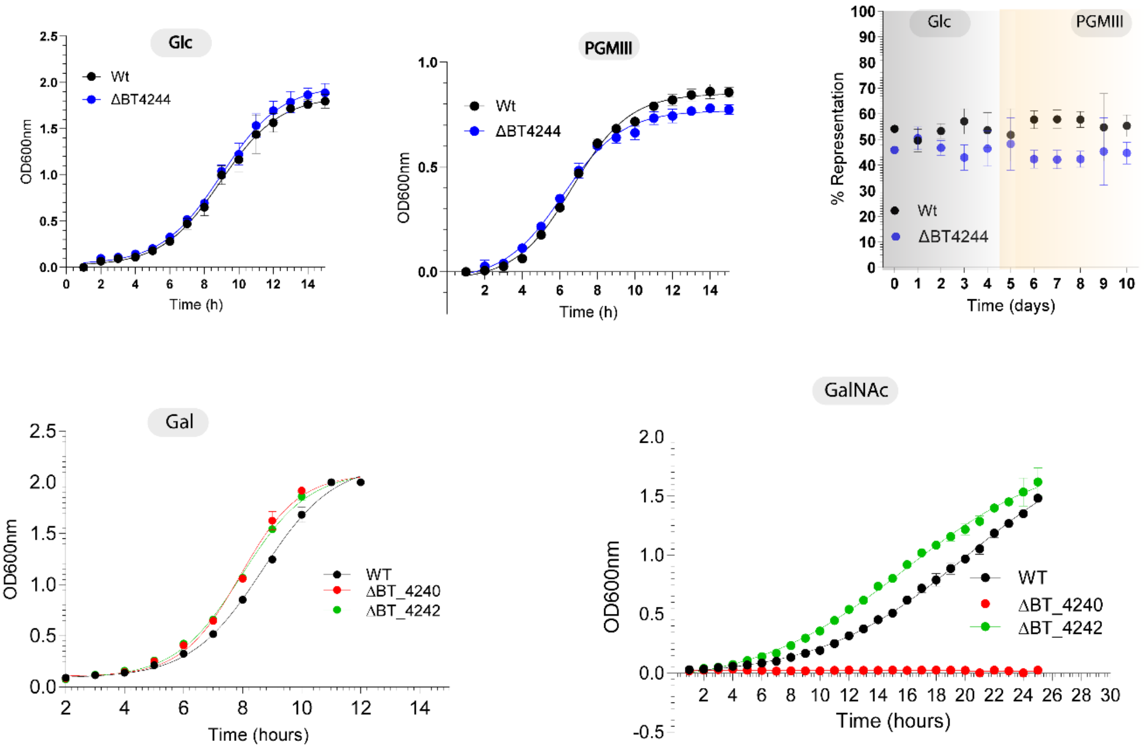

Supplemental Figure 8: Growth curves of *B. theta* wild type and ΔBT4244-M60-like, ΔBT4240-kinase and ΔBT4242 (putative MFS cytoplasmic membrane transporter) mutant strains. Top panel shows growth on minimal media (MM) with glucose or PGMIII as the sole carbon source and on the right shows the results of competitive growth experiments with wild-type vs ΔBT4244 with Glc and then PGMIII as the sole carbon source. Bottom panel shows growth of wild-type as well as ΔBT4240 kinase and ΔBT4242 deletion mutants on MM-Gal or MM-GalNAc as the sole carbon source. Source data are provided as a Source Data file.

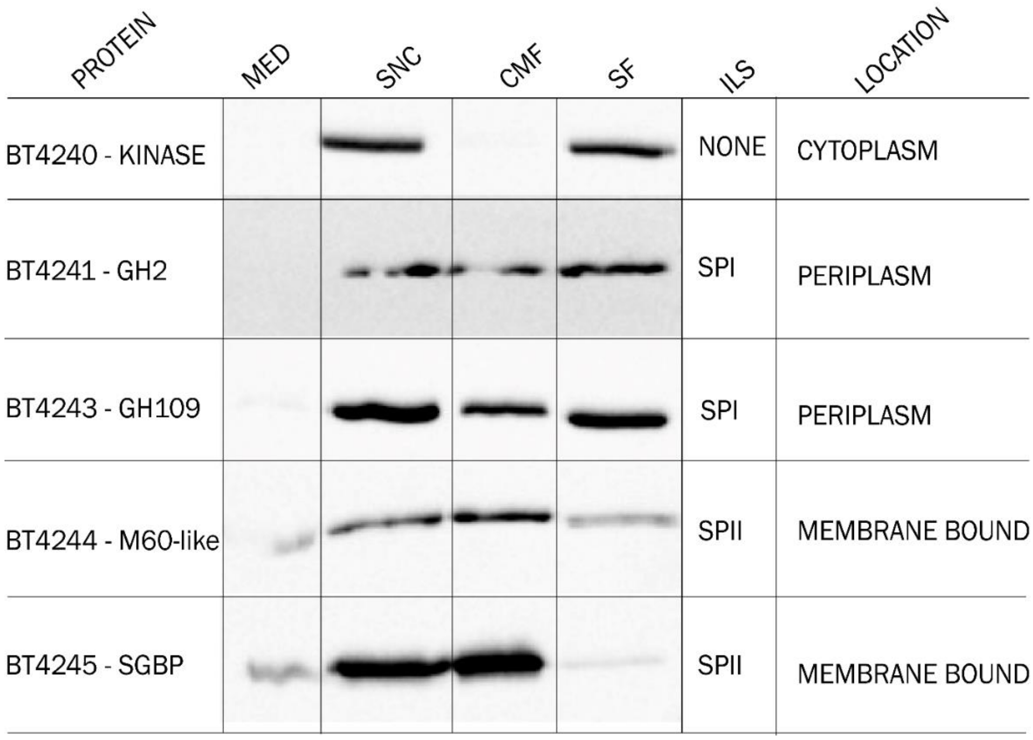

Supplemental Figure 9: Cellular fractionation of PUL BT4240-50 proteins. Strains of *B. theta* expressing C-terminally Flag-tagged proteins were grown on MM-PGMIII and expression and cellular localisation of the tagged proteins analysed by subcellular fractionation and detection using anti-Flag and HRP-conjugated secondary antibodies. MED – spent medium (concentrated to same volume as other fractions), SNC – cell lysate after sonication and centrifugation (cytoplasm and periplasm), CMF – pellet after ultracentrifugation of sonicated cells (membrane fraction), SF – supernatant after ultracentrifugation of sonicated cells (a mix of proteins from various compartments). ILS – in-silico localisation signal based on prediction of signal peptides using LipoP 1.0 server [3]. Note that the ORF of BT4241 in protein databases (e.g. Uniprot accession Q89ZY0) has most likely the wrong predicted N-terminal methionine (see Methods). The most likely correct N-terminal end, with methionine 21 amino acids upstream, corresponds to a strongly predicted SPI. Location column shows the predicted cellular location based on the experimental data shown as well as the following for each considered protein. For the GalNAc kinase it is predicted that the protein is cytoplasmic based on the following evidence: (i) The lack of signal peptide, (ii) The kinase needs ATP for activity and ATP is not present in the periplasm and (iii) BT4240 is absent in membrane fractions (CMF) showing no interaction with the membrane or protein translocation apparatus. For BT4243 and BT4241, the prediction they are periplasmic is based on both proteins having Type I signal peptides and are thus most likely secreted into the periplasm in *Bacteroides* spp,, which is also supported by the evidence that these enzymes are detected in the membrane fraction, consistent with their transport across the cytoplasmic membrane into the periplasm.

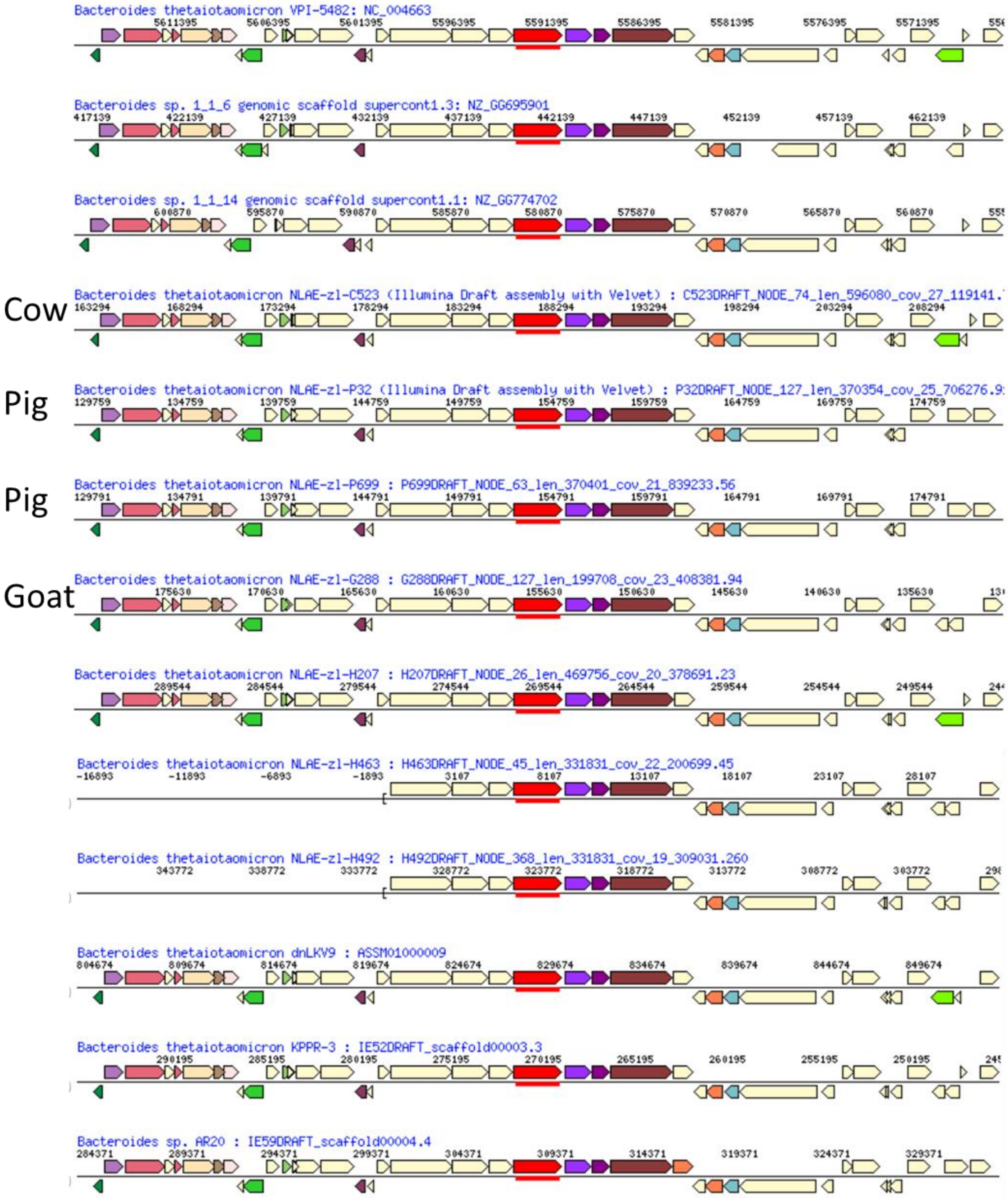

Supplemental Figure 10: Conservation of PUL BT4240-50 across *B. thetaiotaomicron* isolates from humans and animals. A total of 14 annotated *B. theta* genomes encoding identical PULs to BT4240-50 were identified using BlastP with BT4244 as query at the IMG database (identity of ≥99% to BT4244). Of these genomes two are derived from the same strain sequence data and only the original annotation is shown (*B. theta* VPI-5482, NC_004663, top entry). The distinct 13 genomes show the same gene set and order (synteny) compared to *B. theta* VPI-5482. For two genomes the scaffolds covering the set of BT4240-50 homologues are partial and do not include the region encoding the three ECF sigma/anti-sigma regulatory proteins characteristic of the PUL. All but four strains are from humans with the four animal derived strains being NLAE-z1-C523 (cow), NLAE-z1-P32 (pig), NLAE-z1-P699 (pig), NLAE-z1-G288 (goat). The figure was generated with the "Gene Cart Neighborhoods" tool at the IMG database (https://img.jgi.doe.gov/cgi-bin/w/main.cgi).

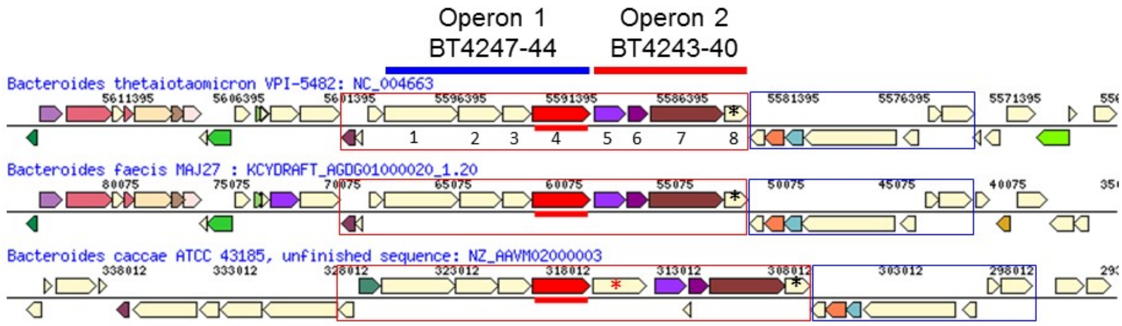

Locus tags for Bt, B. faecis and B.caccae:

1. BT4247 – KCYDRAFT_01778 (97% ID) – BACCAC_01844 (63% ID) – SusC-like
2. BT4246 – KCYDRAFT_01777 (97% ID) – BACCAC_01843 (52% ID) – SusD-like
3. BT4245 – KCYDRAFT_01776 (92% ID) – BACCAC_01842 (26% ID) – SGBP
4. BT4244 – KCYDRAFT_01775 (95% ID) – BACCAC_01840 (63% ID) + BACCAC_01841(52% ID) – M60L
5. BT4243 – KCYDRAFT_01774 (97% ID) – BACCAC_01839 (87% ID) – GH109
6. BT4242 – KCYDRAFT_01773 (98% ID) – BACCAC_01838 (93% ID) – Putative transporter
7. BT4241 – KCYDRAFT_01772 (95% ID) – BACCAC_01836 (86% ID) – GH2
8. BT4240 – KCYDRAFT_01771 (97% ID) – BACCAC_01835 (96% ID) – GalNAc kinase

Supplemental Figure 11: Comparison of gene neighbourhood of *B. thetaiotaomicron* BT4240-50 with related PULs from *B. faecis* and *B. caccae*. In addition to the *B. theta* genomes encoding homologues of PUL BT4240-50, *Bacteroides faecis* MAJ27 and *Bacteroides caccae* ATCC43185 also encode identical (*B. faecis*: KCYDRAFT_01771-81) or very similar, PULs (*B. caccae*: BACCAC_1835-46) (the three red boxes), with the latter characterised by an additional M60L peptidase (BT4244 and homologues are shown in red in the three gene neighbourhoods and the additional M60L homologue in *B. caccae* is indicated by the red star). The BT4240 GalNAc kinase and homologues are indicated by a black star. *B. theta* and *B. faecis* share an identical gene neighbourhood organisation across the majority of the shown genome segment. The genes downstream BT4240 and its corresponding homologues from *B. faecis* and *B. caccae* (indicated by a black stars) are conserved between the three species (blue box). The figure was generated with the "Gene Cart Neighborhoods" tool at the IMG/M database (https://img.jgi.doe.gov/cgi-bin/w/main.cgi). The locus tags, annotations and sequence identity (% values between brackets) between *B. theta* proteins and respectively *B. faecis* and *B. caccae* proteins are listed below the figure.

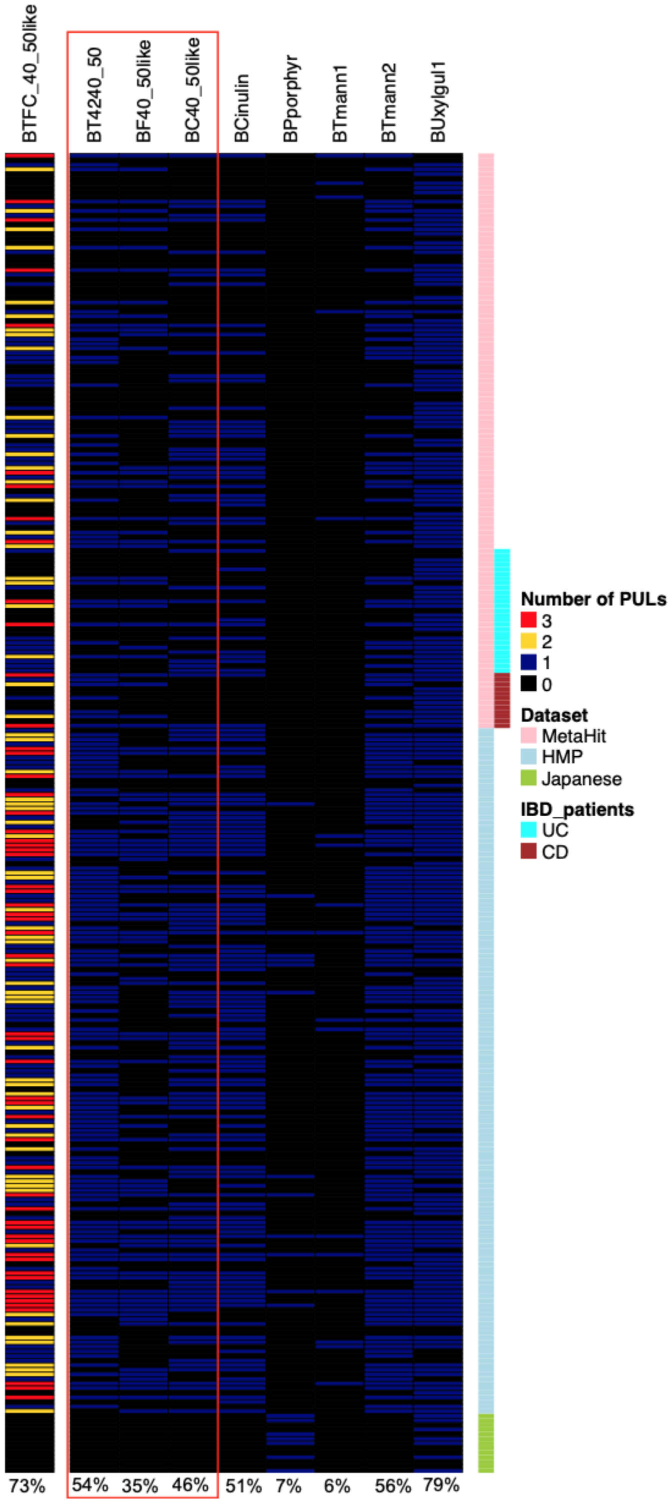

Supplemental Figure 12: Distribution of *B. thetaiotaomicron* PUL BT4240-50 and the related PUL from *B. faecis* and *B. caccae* across human metagenome datasets. The human gut metagenome sequence data from 287 individuals were analysed as described previously [9,10]. The three datasets of human metagenomes were from top to bottom: (i) samples from the MetaHit project [11], (ii) the HMP [12] and (iii) 13 healthy Japanese individuals [13]. Samples from inflammatory bowel disease (IBD) patients among the MetaHit dataset are also indicated - ulcerative colitis (UC) and Crohn's disease (CD). The samples were queried by Blast using DNA sequences from indicated PULs (see Methods section for details). Each blue horizontal line represents a positive sample from a given individual. The first column on the left (BTFC40_50like) summarises the sum of the samples positive for the three

related BT4240-50 PULs (*B. thetaiotaomicron* BT4240_50), KCYDRAFT_01771-81 (*B. faecies*: BF40_50like) and BACCAC_1835-46 (*B. caccae*: BC40_50like) (See Supplementary Fig. 11). The data for these three PULs are highlighted within the red box. The distribution of these three PULs was contrasted with a selection of PULs from various *Bacteroides* species with contrasting frequencies of occurrence across human gut metagenomes: *B. caccae* inulin PUL (BCinulin, common) [14], *B. plebeius* porphyran PUL (BPporphyr, rare outside Japanese) [15]; *B. theta* mannan 1 PUL (BTmann1, rare) and *B. theta* mannan 2 PUL (BTmann2, common) [9]; and *B. uniformis* xyloglycan PUL (BUxylglul1, very common) [16]. The frequency of a given PUL across the 287 samples is shown at the bottom of each column.

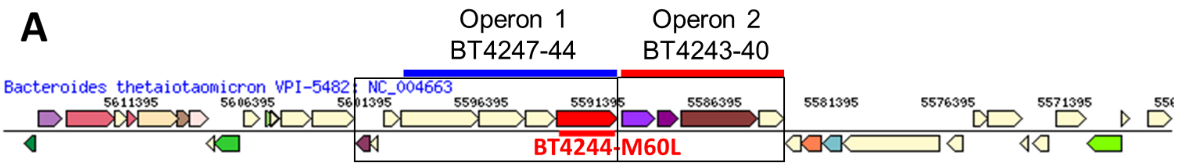

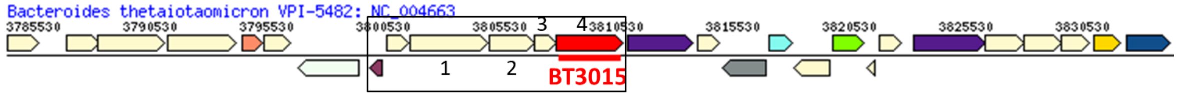

Substrate: host glycans (unknown type, PGM phase 2) (PUL 45 in Martens et al. 2008)

1. BT3012: SusC/RagA family
2. BT3013: RagB/SusD
3. BT3014: DUF4959, (PF16323), [CBM32-like - IPR008979 Galactose-binding domain-like]
4. BT3015: lipoprotein, BACON (PF13004), CBM32 (PF00754). M60L (PF13402)

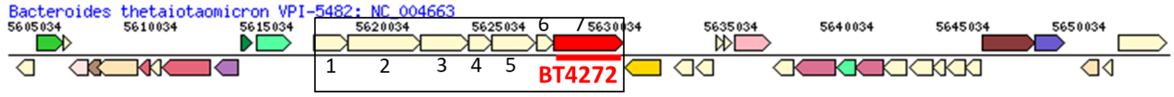

Substrate: unknown (PUL 79 in Martens et al. 2008)

1. BT4266: hypothetical protein, NHL repeat (PF01436)
2. BT4267: SusC/RagA family
3. BT4268: RagB/SusD
4. BT4269: lipoprotein, hypothetical protein, DUF1735 (PF08522), DUF4361 (PF14274)
5. BT4270: lipoprotein, hypothetical protein. 2x BACON (PF13004), CBM32 (PF00754)
6. BT4271: lipoprotein, hypothetical protein, TPR repeats (SSF48452)
7. BT4272: lipoprotein, 2x BACON (PF13004), M60L (PF13402)

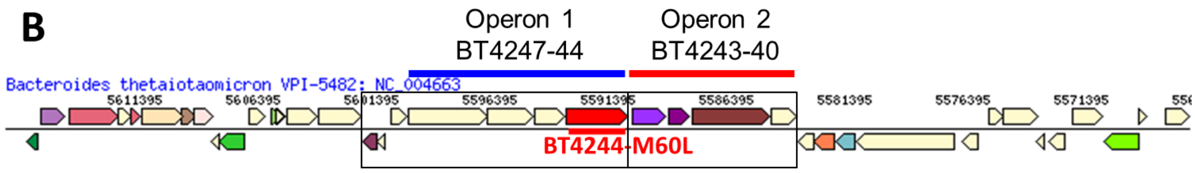

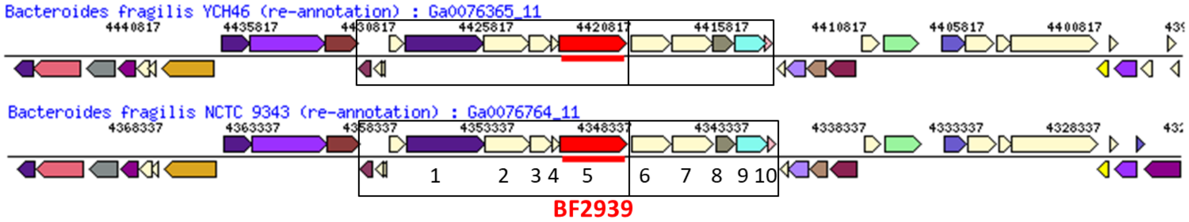

B. fragilis strains YCH46 (re-annotation) - NCTC9342 (re-annotation)

1. Ga0076365_113777 (BF3104) - Ga0076764_113712 (BF2942) : SusC/RagA family
2. Ga0076365_113776 (BF3103) - Ga0076764_113711 (BF2941): RagB/SusD (Pfam PF07980)
3. Ga0076365_113775 (BF3102) - Ga0076764_113710 (BF2940): SGBP: DUF4959 (PF16323), CBM32 (PF00754), domains
4. Ga0076365_113774 - Ga0076764_113709: hypothetical protein
5. Ga0076365_113773 (BF3101) - Ga0076764_113708 (BF2939): 2x BACON (PF13004), CBM32 (PF00754), M60L (PF13402)
6. Ga0076764_113772 (BF3100) - Ga0076764_113707 (BF2938): Phosphofructokinase (PF00365)
7. Ga0076764_113771 (BF3099) - Ga0076764_113706 (BF2937): BNR repeat-like domain-containing protein (PF13088)
8. Ga0076764_113770 (BF3098) - Ga0076764_113705 (BF2936): GH25 (PF01183)
9. Ga0076764_113769 (BF3097) - Ga0076764_113704 (BF2935): hypothetical protein (PF01370 – NAD dependent epimerase/dehydratase family)
10. Ga0076764_113768 (BF3096) - Ga0076764_113703 (BF2934): hypothetical protein (PF14542 – GCN5-related N-acetyl-transferase)

C

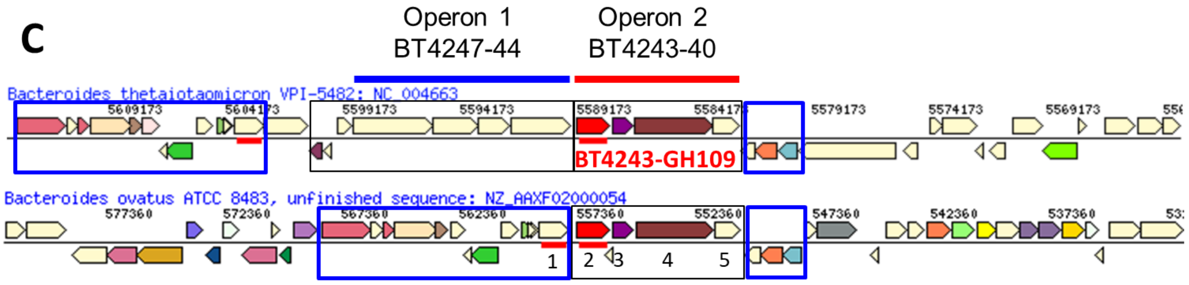

1. BACOVA_05083: GH109 (PF01408) (46%ID with BT4243)
2. BACOVA_05082: GH109 (PF01408) (88%ID with BT4243)
3. BACOVA_05080: PF02588 (DUF161), PF10035 (DUF2179) (93% ID with BT4242)
4. BACOVA_05079: GH2 (89% ID with BT4241)
5. BACOVA_05078: GalNacK, PF01636 Phosphotransferase enzyme family (94% ID with BT4240)

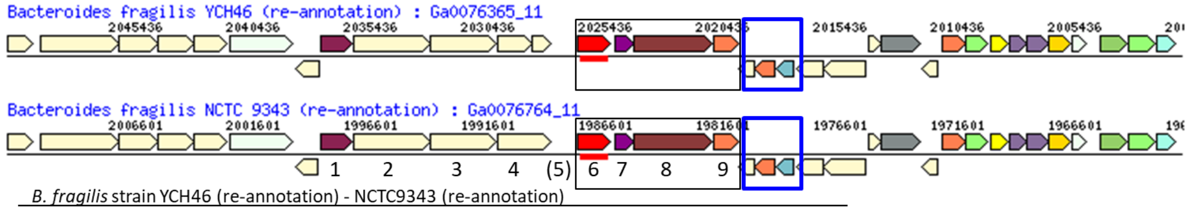

B. fragilis strain YCH46 (re-annotation) - NCTC9343 (re-annotation)

1. Ga0076365_111661 (BF0938) - Ga0076764_111616 (BF0858): MFS transporter, SP family, sugar porter, (PF00083)
2. Ga0076365_111660 (BF0937) - Ga0076764_111615 (BF0857): hypothetical protein, (PF17128: DUF5107, PF13181: TPR repeat)
3. Ga0076365_111659 (BF0936) - Ga0076764_111614 (BF0856): beta-galactosidase, GH2 (PF02837, PF00703)
4. Ga0076365_111658 (BF0935) - Ga0076764_111613 (BF0855): lipoprotein, hypothetical protein
5. BF0934: hypothetical protein; (BF0932 - BF0854: hypothetical protein, gene lost in the re-annotation)
6. Ga0076365_111656 (BF0931) - Ga0076764_111611 (BF0853): GH109 (PF01408) (87%ID with BT4243)
7. Ga0076365_111655 (BF0930) - Ga0076764_111610 (BF0852): PF02588 YitT membrane, (PF10035: DUF2179) (86% ID with BT4242)
8. Ga0076365_111654 (BF0929) - Ga0076764_111609 (BF0851): beta-galactosidase, GH2 (PF02837, PF00703, PF02836, PF16353, PF02929) (77% ID with BT4241)
9. Ga0076365_111653 (BF0928) - Ga0076764_111608 (BF0850): GalNacK, PF01636 Phosphotransferase enzyme family (93% ID with BT4240)

Supplemental Figure 13: Diversity of M60L containing PULs in *B. theta*, and GH109 and GalNAc kinase containing PULs/operons in other *Bacteroides* species. (**A**) Comparison of gene neighbourhood for the three M60L peptidase containing PULs in *B. theta* (M60L gene highlighted and underlined in red). The top panel shows the BT4240-50 PUL with its two known operons indicated [2]. The two additional PULs, shown below, encode one M60L each (BT4272 and BT3015) with the annotation of their neighbouring genes being indicated. ORFs with homologues in BT4240-50 are highlighted in red text. (**B**) Comparison of the PUL BT4240-50 with the unique M60L containing predicted PULs in two *B. fragilis* strains YCH46 and NCTC9343 (M60L gene highlighted in red). Locus tags shown are for the reannotated strains with the original locus tags in parentheses. The putative *B. fragilis* PUL comprises homologous genes to BT4247-44 (*B. theta* operon 2), but in *B. fragilis* the operon (genes labelled 1-5) is adjacent to a set of ORFs encoding predicted carbohydrate active enzymes distinct from those encoded by BT4243-40, suggesting the *B. fragilis* PUL may target a different glycan to BT4240-50. (**C**) Comparison of the *B. theta* operon 1 (BT4240-43), encoding the BT4243-GH109 α-GalNAc'ase (gene highlighted and underlined in red) and BT4240-kinase the GalNAc kinase, (gene highlighted with red stars above the ORF), with related operons in *B. ovatus* and *B. fragilis* (two strains). Although *B. fragilis* can grow on *O*-glycans, *B. ovatus* cannot [17] suggesting *B. ovatus* accesses GalNAc from other sources then mucins. The ORF upstream of GalNAc kinase (94% identity with BT4240 protein) is a homologue but it only share 46% identy with the BT4240 protein. *B. theta* and *B. ovatus* share ORFs upstream and downstream of the BT4240-43 operon and these are boxed in blue. The conserved operon encoding the GH109 and GalNac kinase across different *Bacteroides* species with distinct gene neighbourhoods is consistent with the independent evolution of the two operons from BT4240-50 (BT4244-47 and BT4240-43) with currently only three species noted to have the combination of both (see Supplemental Figure 11).

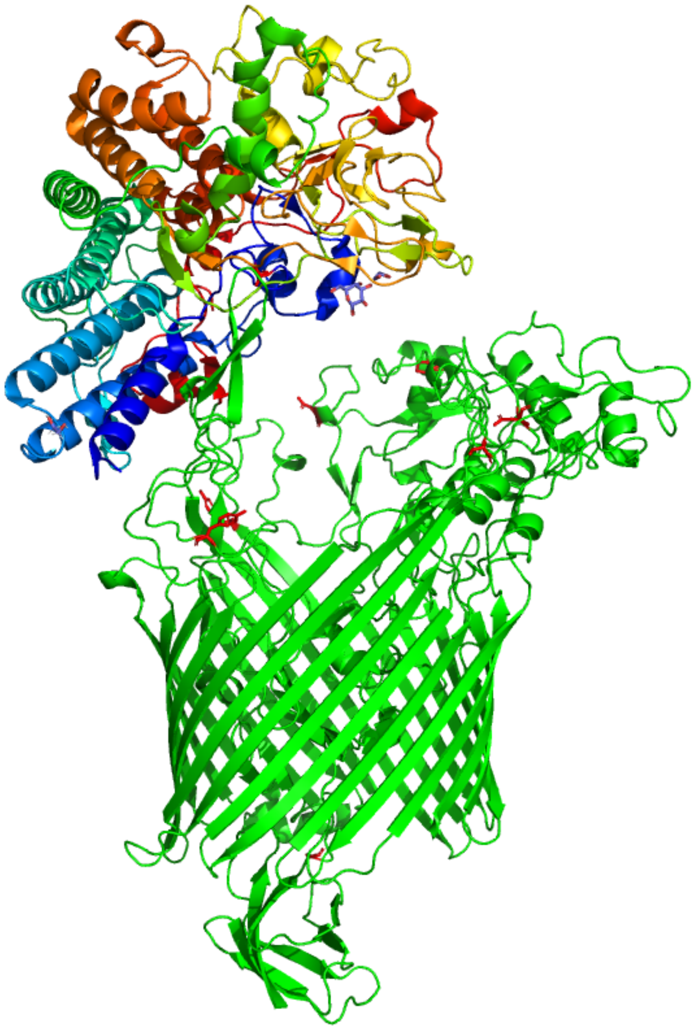

Supplemental Figure 14: Location of main mutations in BT4246-4247 SusC/D in *B. theta* colonising mice fed a western-style diet.

Main sites of mutations identified in *B. theta* geneome from mice fed a low fibre ‘Western style’ diet (WD) where *B. theta* relies more on utilising mucin-derived glycans, compensating for the lack of dietary fibre in the WD [18]. Most of these mutations are in BT4247 SusC at the surface ‘lip’ of the barrel (side chains highlighted as red sticks), suggesting this location plays a key role in modulating the efficiency of mucin glycopeptide uptake. SusC/D model (Alphafold) is the same as shown in Supplemental Fig.4, using the crystal structure of BT4246-SusD and the Alphafold model of BT4247-SusC.

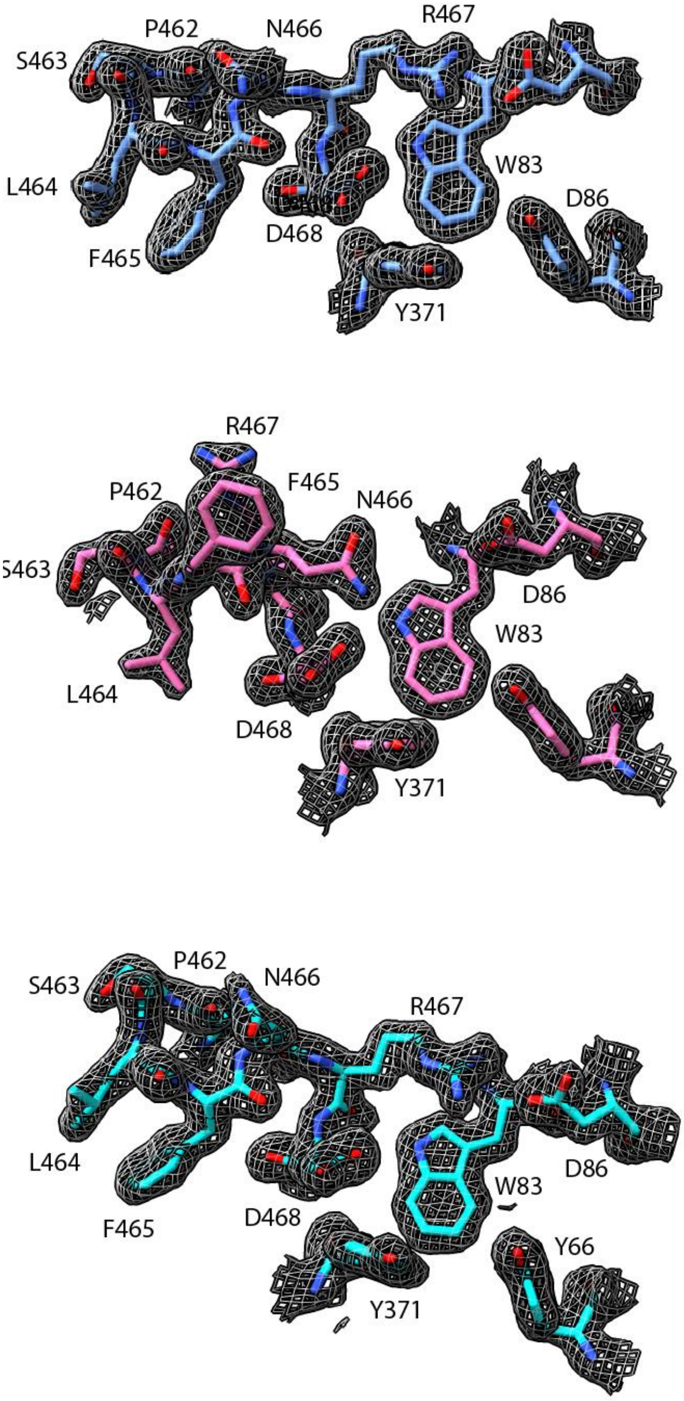

Supplemental Figure 15: A portion of the electron density for each of the BT4246 SusD-like structures solved in this study. Top panel (blue) is mucin oligosaccharide bound structure (PDB 5cjz), middle panel (pink) is SelenoMet (PDB 5ck1) and bottom panel (cyan) is native (PDB 5ck0). All are 2Fo-Fc contoured at 1.2 sigma. The region aroud the ligand biniding site is shown for all.

References

1. Liu, Q.P., et al., *Bacterial glycosidases for the production of universal red blood cells.* Nat Biotechnol, 2007. **25**(4): p. 454-64.
2. Martens, E.C., H.C. Chiang, and J.I. Gordon, *Mucosal glycan foraging enhances fitness and transmission of a saccharolytic human gut bacterial symbiont.* Cell Host Microbe, 2008. **4**(5): p. 447-57.
3. Juncker, A.S., et al., Prediction of lipoprotein signal peptides in Gram-negative bacteria. Protein Sci, 2003. 12(8): p. 1652-62.
4. Massimino, L., et al., *The Inflammatory Bowel Disease Transcriptome and Metatranscriptome Meta-Analysis (IBD TaMMA) framework.* Nat Comput Sci, 2021. **1**(8): p. 511-515.
5. Koropatkin, N.M., et al., *Starch catabolism by a prominent human gut symbiont is directed by the recognition of amylose helices.* Structure, 2008. **16**(7): p. 1105-15.
6. Ren, J., et al., DOG 1.0: illustrator of protein domain structures. Cell Res, 2009. 19(2): p. 271-3
7. Rahfeld, P., et al., An enzymatic pathway in the human gut microbiome that converts A to universal O type blood. Nat Microbiol, 2019. 4(9): p. 1475-1485.
8. Wang, K.C., et al., Insights into the binding specificity and catalytic mechanism of N-acetylhexosamine 1-phosphate kinases through multiple reaction complexes. Acta Crystallogr D Biol Crystallogr, 2014. 70(Pt 5): p. 1401-10.
9. Cuskin, F., et al., Human gut Bacteroidetes can utilize yeast mannan through a selfish mechanism. Nature, 2015. 517(7533): p. 165-169.
10. Larsbrink, J., et al., A discrete genetic locus confers xyloglucan metabolism in select human gut Bacteroidetes. Nature, 2014. 506(7489): p. 498-502.
11. Qin, J., et al., A human gut microbial gene catalogue established by metagenomic sequencing. Nature, 2010. 464(7285): p. 59-65.
12. Human Microbiome Project, C., Structure, function and diversity of the healthy human microbiome. Nature, 2012. 486(7402): p. 207-14
13. Kurokawa, K., et al., Comparative metagenomics revealed commonly enriched gene sets in human gut microbiomes. DNA Res, 2007. 14(4): p. 169-81.
14. Pudlo, N.A., et al., Symbiotic Human Gut Bacteria with Variable Metabolic Priorities for Host Mucosal Glycans. mBio, 2015. 6(6): p. e01282–15.
15. Hehemann, J.H., et al., Transfer of carbohydrate-active enzymes from marine bacteria to Japanese gut microbiota. Nature, 2010. 464(7290): p. 908–12.
16. Larsbrink, J., et al., A discrete genetic locus confers xyloglucan metabolism in select human gut Bacteroidetes. Nature, 2014. 506(7489): p. 498–502.
17. Martens, E.C., et al., Recognition and degradation of plant cell wall polysaccharides by two human gut symbionts. PLoS Biol, 2011. 9(12): p. e1001221.
18. Dapa, T., et al., Diet leaves a genetic signature in a keystone member of the gut microbiota. Cell Host Microbe, 2022. 30(2): p. 183-199 e10

Uncropped scans of blots and gels in supplemental figures:

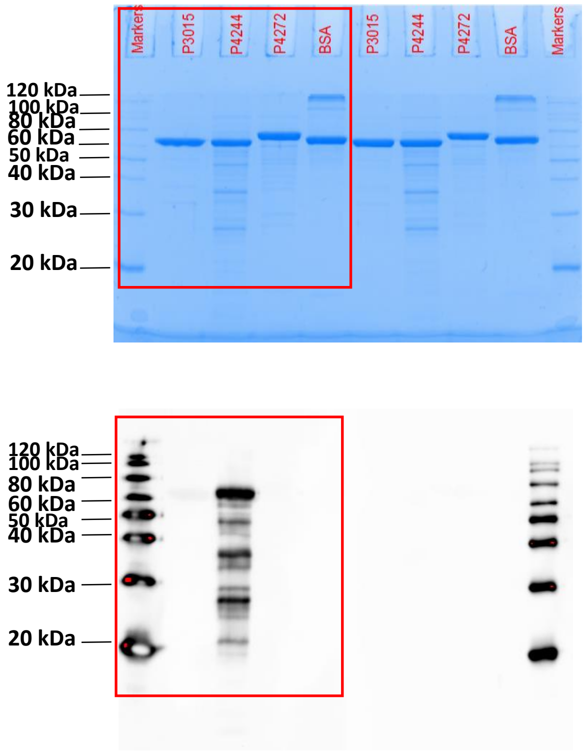

Supplemental Figure 1A: Full/uncropped gel image. Cropped region used for figure is shown in red rectangle

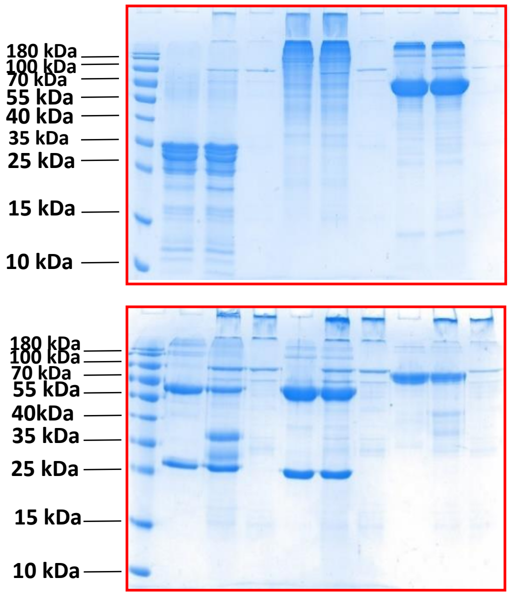

Supplemental Figure 1B: Full/uncropped gel image. Cropped region used for figure is shown in red rectangle

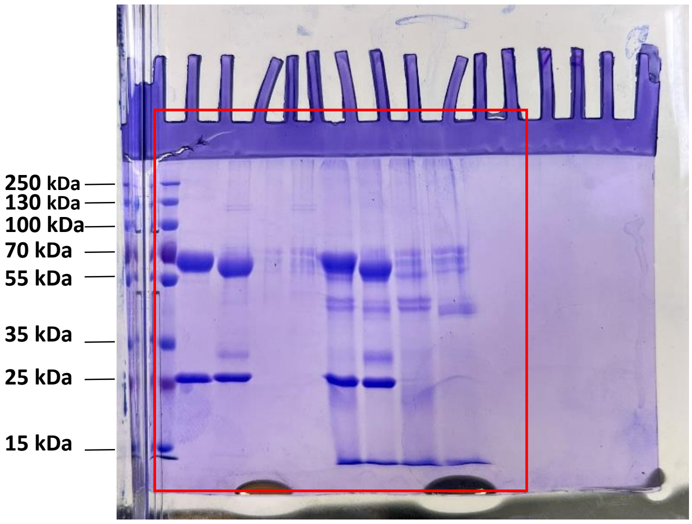

Supplemental Figure 1C: Full/uncropped gel image. Cropped region used for figure is shown in red rectangle

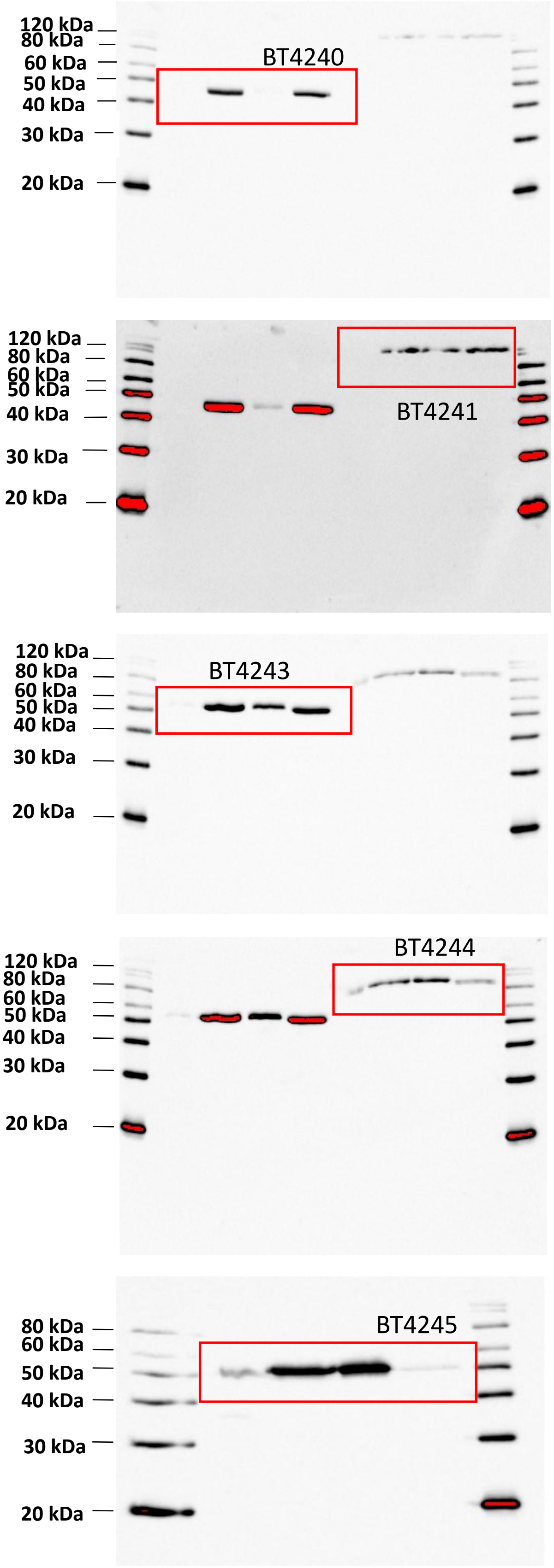

Supplemental Figure 9: Full/uncropped gel image. Cropped region used for figure is shown in red rectangle
